# Association between maternal health service utilization and under-five mortality rate in China and its provinces, 1990–2017

**DOI:** 10.1186/s12884-024-06437-8

**Published:** 2024-04-26

**Authors:** Jingya Zhang, Haoran Li, Bincai Wei, Rongxin He, Bin Zhu, Ning Zhang, Ying Mao

**Affiliations:** 1 School of Public Policy and Administration, Xi’an Jiaotong University, Xi’an, Shaanxi 710049, China; 2 School of Public Health and Emergency Management, Southern University of Science and Technology, Shenzhen, Guangdong 518055 China; 3 Vanke School of Public Health, Tsinghua University, Beijing, 100084 China

**Keywords:** Maternal health service, Under-five mortality rate, China

## Abstract

**Background:**

The United Nations (UN) Sustainable Development Goal − 3.2 aims to eliminate all preventable under-five mortality rate (U5MR). In China, government have made efforts to provide maternal health services and reduce U5MR. Hence, we aimed to explore maternal health service utilization in relation to U5MR in China and its provinces in 1990–2017.

**Methods:**

We obtained data from Global Burden of Disease 2017, China Health Statistics Yearbook, China Statistical Yearbook, and Human Development Report China Special Edition. The trend of U5MR in each province of China from 1990 to 2017 was analyzed using Joinpoint Regression model. We measured the inequities in maternal health services using HEAT Plus, a health inequity measurement tool developed by the UN. The generalized estimating equation model was used to explore the association between maternal health service utilization (including prenatal screening, hospital delivery and postpartum visits) and U5MR.

**Results:**

First, in China, the U5MR per 1000 live births decreased from 50 in 1990 to 12 in 2017 and the average annual percentage change (AAPC) was − 5.2 (*p* < 0.05). Secondly, China had a high maternal health service utilization in 2017, with 96.5% for prenatal visits, 99.9% for hospital delivery, and 94% for postnatal visits. Inequity in maternal health services between provinces is declining, with hospital delivery rate showing the greatest decrease (SII, 14.01 to 1.87, 2010 to 2017). Third, an increase in the rate of hospital delivery rate can significantly reduce U5MR (OR 0.991, 95%CI 0.987 to 0.995). Postpartum visits rate with a one-year lag can reduce U5MR (OR 0.993, 95%CI 0.987 to 0.999). However, prenatal screening rate did not have a significant effect on U5MR.

**Conclusion:**

The decline in U5MR in China was associated with hospital delivery and postpartum visits. The design and implementation of maternal health services may provide references to other low-income and middle-income countries.

**Supplementary Information:**

The online version contains supplementary material available at 10.1186/s12884-024-06437-8.

## Background

Children’s health is widely recognized as a public health priority in every country. The under–five mortality rate (U5MR), which estimates the probability of dying between birth and the fifth birthday (usually expressed per 1000 live births), is a useful indicator that measures not only the level of child health, but also the economy, education, and medical care in a country [1]. Sustainable and Development Goals (SDG) 3.2, set by United Nations (UN), calls for an end to avoidable deaths of children, with all countries aiming to decrease U5MR to at least as low as 25 deaths per 1,000 live births by 2030 [2, 3].

Over the past several decades, the world has recorded remarkable progress on child survival. This global U5MR decreased from 71.2/1000 live births in 2000 to 37.1/1000 live births in 2019 [4]. Although U5MR has been significantly reduced globally, it still falls short of the SDG target. Even some developing countries face poor child survival. Sub-Saharan Africa remains the region with the highest U5MR in the world, 76 deaths/1000 live births, which is equivalent to 1 child in 13 dying before reaching the age of 5 [5]. The U5MR also remains a major public-health issue in some Belt and Road Initiative countries [6]. As one of the few countries that have already achieved the third SDG child health goal, China’s practices deserve further evaluation, both to understand the Chinese experience and to provide lessons for other developing countries undergoing health reform alongside rapid social and economic development.

The U5MR in a region is closely related to the state of socio-economic development [7, 8]. For example, regional gross domestic product (GDP) [9], national educational attainment [10], gender inequality index [11], and health care policies [12] have all been verified to be associated with U5MR. Mosley & Chen [13] propose that all social and economic determinants of child mortality must work through a common set of biological mechanisms or proximate determinants to have an impact on mortality. These proximate determinants include maternal factors, environmental contamination, nutrient deficiency, injury, and personal illness control. For children under five years of age, maternal factors are the most important proximate determinants because, lacking self-awareness, they are more dependent on maternal guidance for nutrient deficiency, injury, and personal illness control. Mothers are the primary care-givers of children under the age of five. Their health-seeking behavior during and after pregnancy tends to influence the chances of child survival during the first five years of life [14–16]. For example, place of delivery [17], birth interval [18], breastfeeding [19], and behavioral habits during pregnancy [20] (e.g., smoking and drinking) can affect the child’s health. In such an association framework, the utilization of maternal health services by mothers has an impact on U5MR that cannot be ignored.

In many countries and regions, maternal health services are considered as an important component of primary health care policies and are closely related to the overall level of economic development of the country. Maternal health services can directly affect a variety of maternal characteristics. Prenatal screening [21] is available for genetic and infectious diseases, continuous monitoring of various health indicators and guidance on various physiological hygiene and nutritional. Hospital delivery is equipped with professional delivery rooms, delivery equipment and midwives, which can effectively eliminate neonatal tetanus [22], etc. The postpartum visits can keep track of the changes in the newborn’s signs and breastfeeding, so that problems can be detected and health guidance can be given [23].

Maternal health services in China have covered prenatal screening, hospital delivery, and postpartum visits. In 2009, China launched a comprehensive health care reform that included maternal health services as part of the basic public health service program, which is provided free of charge by the government. Services related to maternal health [24] in basic public health services include free prenatal screening to promote healthy childbirth and child development for all rural couples, subsidized hospital delivery for rural women, and free postpartum visits by maternal and child doctors at the community health services in their resting places. Postpartum visits are carried out in conjunction with maternal health management, and newborn visits are also carried out to strengthen newborn care guidance. It is the responsibility of mothers, where health facilities are available and accessible, to visit these to receive proper medical care during pregnancy, at delivery and after childbirth to promote good health and preserve the lives of herself and her child. With these reform measures, China has made great strides in improving the health of women and children. Overall, maternal mortality declined substantially and rapidly [25], from 108.7 per 100 000 live births in 1996 to 21.8 per 100 000 live births in 2015, making the deceleration rate 8.5%. After years of effort, the urban–rural disparity of maternal mortality in China has also been greatly narrowed. The maternal mortality between urban and rural areas changed from 1:2.37 in 2000 to 1:1.05 in 2015.

In recent decades, China has seen a dramatic decline in U5MR and a significant increase in maternal health service utilization. Despite these achievements, the effectiveness of maternal health service utilization in reducing under-five mortality has not been measured, so we explored the association between maternal health service utilization and under-five mortality. Determining the relationship between U5MR and prenatal screening, hospital delivery, and postnatal visits is key to planning and implementing interventions. Therefore, this study aimed to determine: (1) the trends of U5MR in various provinces of China. (2) the degree of inequity in maternal health services utilization between provinces and its trends. (3) the association between U5MR and maternal health service utilization in China.

## Methods

### Study design and data sources

We obtained U5MR data from Global Burden of Disease (GBD) 2017, which assess U5MR at the provincial level in China from 1990–2017 [26]. This analysis includes 34 province-level administrative units in China (Xinjiang Production and Construction Corps is excluded). These province-level units consist of 23 provinces, five autonomous regions, four municipalities, and two special administrative regions but are all termed provinces. GBD is a widely used database coordinated by the Institute for Health Metrics and Evaluation [27], with data downloaded from the Global Burden of Disease Results (https://vizhub.healthdata.org/gbd-Results//).

Maternal health service utilization rate data were obtained from the China Health Statistics Yearbook, including prenatal screening rate, hospital delivery rate, and postpartum visits rate. The prenatal screening rate refers to the ratio of the number of mothers who received one or more prenatal checkups to the number of live births during the year, in %. The hospital delivery rate refers to the ratio of the number of live births delivered in institutions qualified for midwifery technology to the number of all live births during the year, in %. The postpartum visits rate refers to the ratio of the number of women who had one or more postpartum visits to the number of live births during the year, in %.

Data on demographic characteristics, socioeconomic status, and health services were obtained from the China Statistical Yearbook, the China Health Statistics Yearbook, and the Human Development Report China Special Edition, and we collected data from 2002 to 2017 based on data availability and completeness, which were used as covariates in our model.

We matched U5MR data, maternal health service utilization data (including prenatal screening rate, hospital delivery rate, and postpartum visits rate.), demographic characteristics data (including years of education per capita, birth rate), socioeconomic status data (including GDP per capita, disposable income per capita, emissions of particulate matter in exhaust gases), and health services data (including total number of health workers) based on two variables: region and year.

### Data analysis

Joinpoint model was used to analyze the trend of U5MR from 1990 to 2017 for the country and each province, respectively. Joinpoint analysis was based on a Poisson regression model, and the optimal number of connection points was selected by a substitution test [28]. In this study, the natural logarithm of U5MR was selected as the response variable and the notification year was used as the independent variable. The annual percent change (APC), average annual percentage change (AAPC) and its 95% Confidence Interval (CI) were reported. If the lower CI of AAPC is above 0, it reveals an uphill tendency of the indicator, and; if the upper CI of AAPC is below 0, it indicates a downward trend of the indicator. Each P-value was found calculated using the Monte Carlo methods, and the overall asymptotic significance level was maintained through a Bonferroni correction. P value of less than 0.05 was considered statistically significant. Additionally, if the confidence interval contains 0, it indicates that the trend of change is not statistically significant.

The UN’s Health Inequity Toolkit HEAT Plus was used to measure the inequity of maternal health services (including prenatal screening, hospital delivery, and postpartum visits) in each province in China, comparing its change between 2010 and 2017. The UN’s Health Inequities Toolkit is available at the website (https://www.who.int/data/inequality-monitor). First, we finished that the uploaded data passed validation using HEAT Plus template and validation before performing analysis. The UN’s Health Inequity Toolkit can export a range of health inequity indicators such as Difference (D), Absolute concentration index (ACI), Population attributable fraction (PAF), Population attributable risk (PAR). Ratio (R), Relative concentration index (RCI), and other health inequity indicators. This study additionally calculated the slope index of inequality (SII) to measure the absolute difference between the highest and lowest levels of maternal health care between provinces. The x-axis represents the relative order of each province (weighted order after population weighting), and the y-axis represents the U5MR for each province. The relative order of provinces is the midpoint of the cumulative population fraction. The SII is the slope of the regression line of y versus x. The determination of the relative order in this study included the following steps: first, all provinces were ranked from lowest to highest according to the Human Development Index (HDI); then, the relative order of the subgroup was determined based on the population fraction of each province. For all measures of inequity, the lower the value, the more equitable it is.

To assess the association between maternal health service utilization (including prenatal screening, hospital delivery, and postpartum visits) and U5MR, we applied the generalized estimating equation model, a widely used linear model for longitudinal data analysis with repeated measures over time [29]. The generalized estimating equation (GEE) model used a gamma distribution and log-link function to control for the skewed nature of mortality. The dependent variable refers to ln (U5MR). In multivariable models, we controlled for year, years of schooling per capita, GDP per capita, disposable income per capita, birth rate, total number of health workers and emissions of particulate matter in exhaust gas. To strengthen the longitudinal analysis, we also examined the lagged effects [30–32] of prenatal visits and postnatal visits. To test the robustness of the results, in sensitivity analyses we replaced gamma distribution with Poisson distribution, gaussian distribution, and negative binomial distribution for the GEE model analysis, respectively. STATA version 13.0 was used in this study, and statistical significance was attributed to P values < 0.05. All figures were drawn by using OriginPro (version 2023b).

## Results

Table 1 showed the U5MR in China and each province in 2017 and the trend from 1990 to 2017. The overall U5MR in China in 2017 was 12 per 1000, which was in a decreasing trend from 1990 to 2017 (AAPC − 5.2, 95%CI −5.3 to −5.1). Most provinces met the SDG 3 of reducing U5MR to less than 25 per 1,000 live births, with only two provinces, Xinjiang (28 per 1000) and Tibet (36 per 1000), which were in remote western regions, still having relatively high U5MR. Beijing, as the capital city, was rich in various social resources and has the lowest U5MR of 5per 1000. Some east economically developed regions also had relatively low U5MR, such as Shanghai (7 per 1000), Jiangsu (7 per 1000), Zhejiang (7 per 1000), Guangdong (7 per 1000), Fujian (7 per 1000), and Liaoning (7 per 1000). Each province in China had seen a decrease in U5MR from 1990 to 2017. The central and western regions were decreasing at a faster rate, such as Guizhou (AAPC − 6.7, 95%CI −6.8 to −6.6). The eastern regions were decreasing at a relatively slow rate, such as Tianjin (AAPC − 2.8, 95%CI −3.0 to −2.6).

**Table 1:** Log-transformed joinpoint trends of U5MR in China and its provinces, 1990–2017

Sex	Trend 1		Trend 2		Trend 3		Trend 4		Trend 5		2017 U5MR	1990–2017 AAPC (95%CI)
	Years	APC	Years	APC	Years	APC	Years	APC	Years	APC		
China	1990–1999	−3.6*	1999–2012	−6.6*	2012–2015	−2.3*	2015–2017	−7.2*			12	−5.2*(−5.3~−5.1)
Beijing	1990–1998	−2.1	1998–2012	−4.7	2012–2015	0.8	2015–2017	−8.2*			5	−3.6*(−4.0~−3.2)
Tianjin	1990–1998	−1.4*	1998–2017	−3.4*							8	−2.8*(−3.0~−2.6)
Hebei	1990–2001	−1.3*	2001–2017	−4.3*							13	−3.1*(−3.2~−3.0)
Shanxi	1990–1998	−1.9*	1998–2011	−5.1*	2011–2017	−3.6*					12	−3.8*(−3.9~−3.7)
Inner Mongolia	1990–1998	−3.0*	1998–2011	−5.3*	2011–2014	−2.5*	2014–2017	−6.4*			13	−4.4*(−4.5~−4.3)
Liaoning	1990–2003	−1.4*	2003–2012	−5.8*	2012–2017	−3.1					7	−3.2*(−3.5~−3.0)
Jilin	1990–2017	−3.8*									9	−3.8*(−3.9~−3.7)
Heilongjiang	1990–1999	−3.3*	1999–2005	−1.6	2005–2017	−3.9*					12	−3.2*(−3.3~−3.1)
Shanghai	1990–2006	−3.6*	2006–2009	0.1	2009–2017	−3.3*					7	−3.1*(−3.4~−2.8)
Jiangsu	1990–2000	−4.2*	2000–2012	−7.3*	2012–2015	−0.9	2015–2017	−6.7*			7	−5.4*(−5.6~−5.2)
Zhejiang	1990–1998	−2.8*	1998–2017	−6.1*							7	−5.1*(−5.3~−4.9)
Anhui	1990–1999	−4.4*	1999–2017	−5.6*							12	−5.2*(−5.3~−5.1)
Fujian	1990–1998	−2.9*	1998–2017	−5.8*							7	−5.0*(−5.1~−4.8)
Jiangxi	1990–1998	−4.4*	1998–2010	−6.6*	2010–2017	−5.0*					16	−5.5*(−5.6~−5.5)
Shandong	1990–1999	−2.0*	1999–2004	−4.4*	2004–2009	−7.1*	2009–2017				10	−4.1*(−4.2~−3.9)
Henan	1990–1993	−2.1	1993–1999	−5.1*	1999–2010	−7.1*	2010–2017	−3.9*			13	−5.3*(−5.4~−5.1)
Hubei	1990–2012	−5.2*	2012–2017	−3.6*							12	−4.9*(−5.1~−4.7)
Hunan	1990–1999	−4.4*	1999–2010	−8.1*	2010–2017	−4.8*					9	−6.0*(−6.2~−5.9)
Guangdong	1990–1999	−2.3*	1999–2011	−6.4*	2011–2017	−4.0*					7	−4.5*(−4.7~−4.3)
Guangxi	1990–1998	−2.2*	1998–2004	−5.9*	2004–2012	−7.7*	2012–2015	−2.1*	2015–2017	−7.2*	12	−5.0*(−5.2~−4.9)
Hainan	1990–2004	−2.7*	2004–2011	−5.6*	2011–2017	−4.2*					16	−3.8*(−3.9~−3.6)
Chongqing	1990–2000	−4.3*	2000–2010	−6.9*	2010–2015	−4.4*	2015–2017	−6.8*			13	−5.5*(−5.6~−5.4)
Sichuan	1990–2001	−4.8*	2001–2017	−6.2*							15	−5.7*(−5.9~−5.4)
Guizhou	1990–1996	−4.6*	1996–2000	−5.9*	2000–2012	−8.5*	2012–2017	−5.7*			18	−6.7*(−6.8~−6.6)
Yunnan	1990–1998	−4.8*	1998–2008	−7.6*	2008–2017	−5.7*					17	−6.1*(−6.2~−6.0)
Tibet	1990–1998	−3.3*	1998–2011	−5.2*	2011–2015	−3.0*	2015–2017	−6.2*			36	−4.4*(−4.5~−4.3)
Shaanxi	1990–2000	−3.0*	2000–2005	−4.5*	2005–2012	−7.5*	2012–2017	−4.2*			15	−4.7*(−4.8~−4.6)
Gansu	1990–1998	−2.9*	1998–2008	−5.6*	2008–2011	−6.8*	2011–2015	−4.7*	2015–2017	−8.5*	20	−5.0*(−5.1~−5.0)
Qinghai	1990–1998	−4.3*	1998–2012	−6.1*	2012–2015	−3.3*	2015–2017	−8.2*			21	−5.4*(−5.5~−5.3)
Ningxia	1990–2000	−4.5*	2000–2005	−6.2*	2005–2013	−6.9*	2013–2017	−5.6*			16	−5.7*(−5.7~−5.6)
Xinjiang	1990–1998	−3.8*	1998–2017	−4.5*							28	−4.3*(−4.4~−4.2)
Hong Kong	1990–2015	−4.8	2015–2017	−21.9*							1	−6.2*(−7.4~−4.4)
Macau	1990–2017	−5.6*									2	−5.6*(−6.1~−5.2)
Taiwan	/											/

*Notes* AAPC, Average annual percent change; APC, Annual percent change; CI, confidence interval; NA, not applicable. *Significantly different from zero, P value < 0.05

**Fig. 1:**
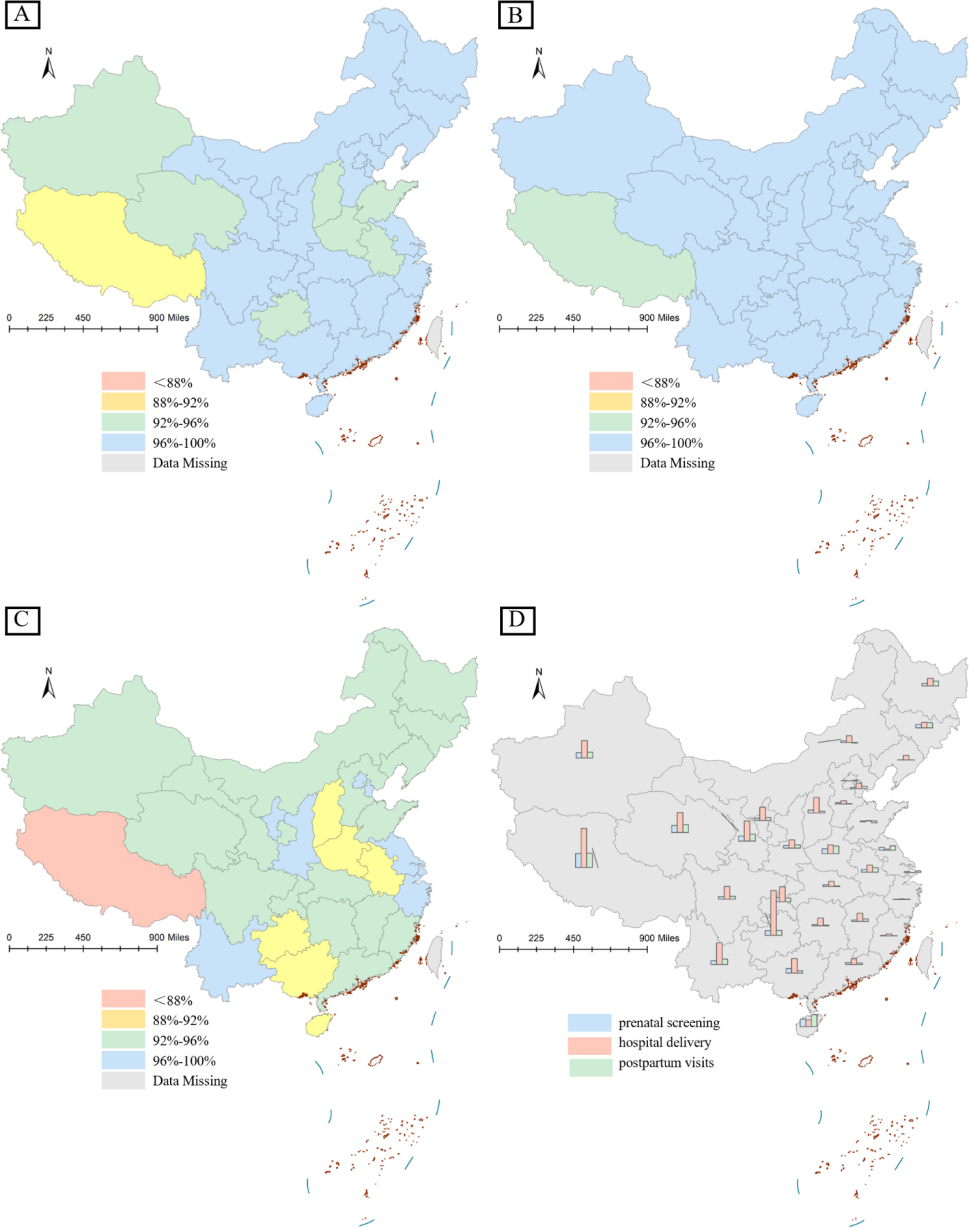
Maternal health service utilization rate by province in China, 2002–2017. *Notes* (**A**) Prenatal screening rates by province in China, 2017; (**B**) Hospital delivery rates by province in China, 2017; (**C**) Postnatal visits rates by province in China, 2017; (**D**) Average annual change in prenatal screening rate, hospital delivery rate, and postpartum visits rate by province, China, 2002–2017

Figure 1 showed the maternal health service utilization in each province from 2002 to 2017. Considering prenatal screening, the prenatal screening rate in China was 96.5% in 2017, and the prenatal screening rate was generally high in all provinces, with only Tibet had a low prenatal screening rate of 89.5%. Most provinces were seeing an increase in the rate of prenatal screening, especially at a faster average annual growth rate in some western provinces, such as Tibet (2.52%) and Qinghai (1.29%). It was worth noting that Hainan, as an eastern city, also had a relatively high average annual growth rate of 1.32%. As for the hospital delivery, the hospital delivery rate in China was 99.9% in 2017, with many provinces reaching 100% hospital delivery rate. Most provinces had a hospital birth rate of over 96%, with only Tibet having a relatively low rate of 92.5%. The hospital delivery showed varying degrees of increase in each province from 2002 to 2017, with the fastest annual growth rates in some western provinces, such as Guizhou (8.06%) and Tibet (7.05%). The average annual rate of change in hospital delivery rates was the fastest growing, much higher than prenatal screening and postpartum visits. Judging from postpartum visits, the postpartum visit rate in China was 94% in 2017. Similarly, Tibet had the lowest postpartum visits rate of 83.4%. Most provinces achieved postpartum visits above 92%, with some western provinces such as Hainan (89.7%), Henan (89.9%), Anhui (90%), Guangxi (90.2%), Guizhou (91.3%), and Shanxi (91.9%) having relatively low postpartum visits rates. Most provinces were seeing a rising trend in postpartum visits rate from 2002 to 2017, especially Tibet and Hainan with a faster rate of increase, with an average annual growth rate of 2.57% and 2.14%, respectively.

**Fig. 2:**
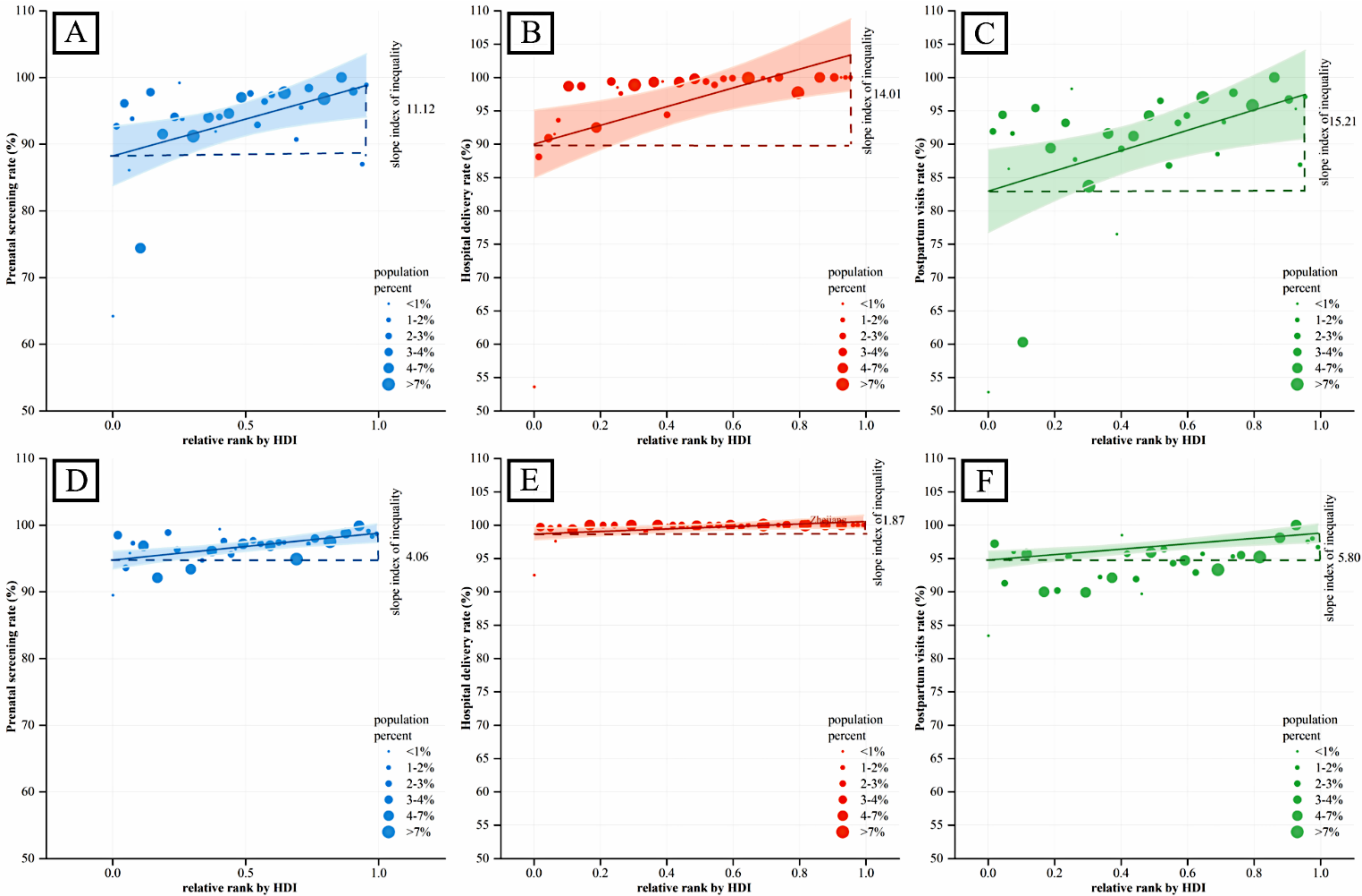
The SII of maternal health services by province in China. *Notes* (**A**) The SII of prenatal screening by province in China, 2017; (**B**) The SII of hospital delivery by province in China, 2017; (**C**) The SII of postpartum visits by province in China, 2017; (**D**) The SII of prenatal screening by province in China, 2010; (**E**) The SII of hospital delivery by province in China, 2010; (**F**) The SII of postpartum visits by province in China, 2010

Table 2; Fig. 2 showed the inequity of maternal health services in each province in China. Looking at prenatal screening, the values of the indicators measuring the inequity of prenatal screening in China, including D, ACI, PAF, R, RCI, and SII, had all been decreasing, especially SII decreased from 11.12 in 2010 to 4.06 in 2017. From hospital delivery, the values of each inequity indicator, including D, ACI, PAF, R, RCI, and SII, were also decreasing, especially SII from 14.01 in 2010 to 1.87 in 2017. Similarly, the values of each inequity indicator for postpartum visits, including D, ACI, PAF, R, RCI, and SII, had decreased, especially SII from 15.21 in 2010 to 5.80 in 2017.

**Table 2:** Inequities in maternal health services by province in China

Summary measure name	Dimension	Prenatal screening		Hospital delivery		Postpartum visits	
		2010	2017	2010	2017	2010	2017
Difference (D)	HDI	34.7	9.2	46.4	7.5	44.3	13.3
Absolute concentration index (ACI)	HDI	1.6	0.6	1.1	0.1	2.4	0.8
Population attributable fraction (PAF)	HDI	4.8	2.1	2.1	0.1	6.3	2.4
Population attributable risk (PAR)	HDI	4.5	2	2	0.1	5.7	2.3
Ratio (R)	HDI	1.5	1.1	1.9	1.1	1.8	1.2
Relative concentration index (RCI)	HDI	1.6	0.6	1.2	0.1	2.6	0.9

*Notes* HDI, Human Development Index

Table 3 presented the association between maternal health service utilization and U5MR. In multivariate generalized estimating equation models, a negative association was observed between hospital delivery and U5MR (OR 0.991, 95%CI 0.987 to 0.995). The association of prenatal screening and postnatal visits with U5MR was not significant. Tables 4 and 5 further demonstrated the lagged effect of prenatal screening and postnatal visits on U5MR. The association between the previous year’s prenatal visit rate and the current year’s U5MR was not significant, however, the association between postpartum visits in the previous year and U5MR in the current year was significant (OR 0.993, 95%CI 0.987 to 0.999). The robustness results show that the significance and direction of the coefficients in the model were consistent we replaced gamma distribution with Poisson distribution, gaussian distribution, and negative binomial distribution for the GEE model analysis, which indicated that the estimates and results of the association between the dependent and independent variables in this study were robust and reliable.

**Table 3:** The association between maternal health service utilization and U5MR in China

Characteristic	Model 1			Model 2			Model 3			Model 4		
	Gamma distribution			Negative binomial distribution			Gaussian distribution			Poisson distribution		
	OR	95%CI		OR	95%CI		OR	95%CI		OR	95%CI	
Prenatal screening	1.000	0.988	1.012	1.000	0.988	1.012	1.001	0.992	1.010	1.001	0.990	1.012
Hospital delivery	0.991*	0.987	0.995	0.991*	0.987	0.995	0.994*	0.992	0.997	0.993*	0.990	0.996
Postpartum visits	1.002	0.993	1.011	1.002	0.993	1.011	1.000	0.991	1.008	1.001	0.993	1.009
Years of schooling per capita	0.925	0.841	1.017	0.926	0.841	1.018	0.943	0.864	1.030	0.935	0.854	1.024
GDP per capita	0.995*	0.991	1.000	0.995*	0.991	1.000	0.996	0.991	1.001	0.996*	0.991	1.000
Birth rate	1.058*	1.020	1.097	1.058*	1.020	1.097	1.058*	1.018	1.100	1.059*	1.020	1.100
Disposable income per capita	0.989	0.977	1.002	0.988	0.976	1.001	0.980*	0.966	0.993	0.983*	0.971	0.996
Total number of health workers	0.999*	0.999	1.000	0.999*	0.999	1.000	0.999*	0.999	1.000	0.999*	0.999	1.000
Emissions of particulate matter in exhaust gas	1.000	0.999	1.002	1.000	0.999	1.002	1.000	0.998	1.001	1.000	0.999	1.001

*Notes* (1) OR, odds ratio, (2) CI, confidence interval, (3) *P < 0.05

**Table 4:** The association between lagged effects of prenatal screening and U5MR in China

Characteristic	Model 5			Model 6			Model 7			Model 8		
	Gamma distribution			Negative binomial distribution			Gaussian distribution			Poisson distribution		
	OR	95%CI		OR	95%CI		OR	95%CI		OR	95%CI	
Prenatal screening	1.002	0.991	1.013	1.002	0.991	1.013	1.003	0.994	1.012	1.003	0.993	1.013
Prenatal screening in the previous year	0.996	0.989	1.003	0.996	0.989	1.003	0.996	0.991	1.000	0.995	0.989	1.002
Hospital delivery	0.991*	0.987	0.995	0.991*	0.987	0.995	0.994*	0.992	0.997	0.993*	0.990	0.996
Postpartum visits	1.004	0.993	1.015	1.004	0.993	1.015	1.001	0.992	1.011	1.003	0.993	1.013
Years of schooling per capita	0.932	0.845	1.029	0.933	0.845	1.030	0.951	0.872	1.037	0.943	0.860	1.034
GDP per capita	0.995*	0.991	1.000	0.995*	0.991	1.000	0.996	0.991	1.001	0.996	0.991	1.000
Birth rate	1.058*	1.019	1.098	1.058*	1.019	1.098	1.061*	1.017	1.106	1.060*	1.019	1.103
Disposable income per capita	1.058	0.976	1.001	0.987	0.975	1.000	0.979*	0.965	0.993	0.982*	0.970	0.995
Total number of health workers	1.058*	0.999	1.000	0.999*	0.999	1.000	0.999*	0.999	1.000	0.999*	0.999	1.000
Emissions of particulate matter in exhaust gas	1.058	0.999	1.002	1.000	0.999	1.002	1.000	0.998	1.001	1.000	0.999	1.001

*Notes* (1) OR, odds ratio; (2) *P < 0.05; (3) CI, confidence interval

**Table 5:** The association between lagged effects of postnatal visits and U5MR in China

Characteristic	Model 9			Model 10			Model 11			Model 12		
	Gamma distribution			Negative binomial distribution			Gaussian distribution			Poisson distribution		
	OR	95%CI		OR	95%CI		OR	95%CI		OR	95%CI	
Prenatal screening	0.999	0.987	1.012	0.999	0.987	1.012	1.002	0.992	1.012	1.001	0.989	1.012
Hospital delivery	0.991*	0.986	0.995	0.991*	0.986	0.995	0.994*	0.991	0.996	0.993*	0.990	0.996
Postpartum visits	1.009	0.998	1.021	1.009	0.998	1.021	1.004	0.995	1.013	1.007	0.997	1.017
Postpartum visits in the previous year	0.993*	0.987	0.999	0.993*	0.987	0.999	0.995*	0.992	0.998	0.994*	0.989	0.999
Years of schooling per capita	0.933	0.846	1.028	0.934	0.847	1.029	0.948	0.870	1.033	0.942	0.860	1.032
GDP per capita	0.996*	0.991	1.000	0.996*	0.991	1.000	0.996	0.991	1.001	0.996	0.992	1.000
Birth rate	1.057*	1.019	1.097	1.058*	1.019	1.098	1.060*	1.017	1.105	1.059*	1.018	1.102
Disposable income per capita	0.988	0.975	1.000	0.987*	0.975	1.000	0.978*	0.964	0.992	0.982*	0.969	0.995
Total number of health workers	0.999*	0.999	1.000	0.999*	0.999	1.000	0.999*	0.999	1.000	0.999*	0.999	1.000
Emissions of particulate matter in exhaust gas	1.000	0.999	1.002	1.000	0.999	1.002	1.000	0.998	1.001	1.000	0.999	1.001

*Notes* (1) OR, odds ratio; (2) *P < 0.05; (3) CI, confidence interval

## Discussion

The U5MR in China was declining, the utilization rate of maternal health services was increasing, and inequalities in maternal health services between provinces were slowly narrowing. The experience of hospital delivery and postpartum visits, both of which had been shown to have a significant impact on the reduction of under-five mortality, might serve as a reference for other countries.

### Changes in U5MR

According to GBD2017 results the U5MR is declining in China and its provinces, which means that child health outcomes are being improved. At the provincial level, only two provinces, Xinjiang (36 per 1000) and Tibet (28 per 1000), did not meet the SDG target. But looking at time trends, the U5MR in Xinjiang and Tibet are on a steady decline, proving that child health outcomes in these areas are getting better. Tibet is in remote areas of China, where the land is vast and the people are sparse, making it more difficult to provide maternal health services as part of basic public health services, but the coverage rate has been increasing, so the health of maternal and child is improving. In some remote rural areas of Tibet, there is still a shortage of human resources for health and health infrastructure [33], and the quality of existing maternal health service provision needs to be improved [34], which can lead to poor maternal and child health outcomes. We should adhere to The Pregnancy and Village Outreach Tibet program [35] and continue to provide families with maternal-newborn health education, skills training, and resources. However, the utilization of maternal health services in Xinjiang is at normal values, which is not quite consistent with its significantly low U5MR. Other factors influencing child health outcomes in Xinjiang still need to be further explored. Some environmental influences can be considered, such as the long summers and low economic performance in some areas of Xinjiang, which are key factors in the high rate of child drowning mortality [36]. In addition, the analysis from intra-provincial suggests that we should also pay attention to U5MR in special populations. At the intra-provincial level. A study [37] based in Zhejiang Province showed that the U5MR among the migrant population was more than twice that of native children (7.82 per 1,000 to 3.89 per 1,000). A study [38] from Henan province shows that although U5MR have declined in recent years, U5MR remain high in rural areas. A study [39] from Sichuan showed that there are ethnic disparities in pneumonia-specific mortality rates among children under 5 years of age in Sichuan, with emphasis on child health in minority counties.

### Inequalities and current status of maternal health services utilization rate

We found that maternal health services utilization is becoming better in every province in China, with prenatal screening rate, hospital delivery rate and postpartum visits rate all gradually increasing, which is consistent with the previous studies [40]. In terms of inequity, the inequity in maternal health services utilization between provinces is decreasing, and the gap in prenatal screening rates, hospital delivery rates, and postpartum visits rates is gradually narrowing across provinces. One of the possible reasons for this is that as Chinese women become more educated, more and more families are paying more attention to maternal and child health, more willing to invest in health care, and increase their use of health care services [41]. At the same time, the country is vigorously promoting fourteen basic public health services, of which maternal health is one of the crucial ones. As a public service, the government invests funds and various administrative efforts to encourage the residents to utilize maternal health services. The dual effect has led to more women taking the initiative to participate in maternal health services.

In particular, the rate of hospital delivery is the highest coverage among the three maternal health services, even reaching 100% hospital delivery of pregnant women in many provinces. There are also a variety of reasons why hospital delivery, a maternal health service, is doing best. One possible reason for this is that incentives vary across service provision. The prenatal screening and postpartum visits provided by primary health care institutions free of charge are a service incentive, while the government provides direct subsidies for hospital delivery as a monetary incentive [42], prompting more rural women to be more willing to take advantage of hospital delivery services, which is one reason why hospital delivery services are better implemented. Compared to service incentives, monetary incentives are more likely to influence people’s behavioral choices [43].

### U5MR and maternal health services utilization rate

We found that hospital delivery in maternal health services significantly reduced U5MR, which is consistent with previous studies [44, 45]. Studies [46, 47] from different regions have shown that neonatal disorders remained the leading cause of death in children younger than 5 years, and the proportion of U5MR occurring in the neonatal period is increasing [48]. Hospital delivery reduce U5MR by reducing neonatal mortality substantially. Medical facilities usually have specialized maternal and child doctors and a more childbirth-friendly environment, which means that the mother is face-to-face with a specialized doctor, whose midwifery practices and resuscitation of emergencies make the improvement in the health of the mother and child more intuitive for everyone. The opposite of hospital delivery is home delivery. Home delivery in the Chinese context is usually performed by acquaintances who have experience in delivering babies, the midwives do not have expertise in childbirth, and the families do not have a safe environment for delivery, resulting in problems such as neonatal asphyxia [49] infection and tetanus in many home births, which will result in the death of the baby. From the international experience, a meta-analysis [50] provides the strongest evidence so far that hospital delivery can, after all, be beneficial to newborn babies. The research from several industrialized countries (the United States, Canada, Australia, Sweden, the Netherlands, and Switzerland), with a total sample of 500,000 newborns, had shown that planned home births to healthy and low-risk mothers compared with planned hospital births in the same group of women doubled the risk of neonatal deaths (0.2% vs. 0.09%). However, this is not absolute, and it is beneficial in some cases to deliver at home. Studies [51] from countries such as Australia, the Netherlands, and United Kingdom show that home birth can provide advantages to the mother and the newborn. It needs to be provided with sufficient material means, and should be attended by trained and accredited professionals, and needs to be perfectly coordinated with the hospital obstetrics and neonatology units, in order to guarantee its safety. However, in China, there are no safety data or sufficient scientific evidence to support home births at present.

Prenatal screening and postpartum visits are more about health guidance, a role that has long-term effects [44] and does not improve maternal and infant health outcomes as quickly and intuitively as hospital births. We validated the lagged effect of prenatal visits and postnatal visits on child health outcomes. The effect of a one-year lag in prenatal visits on U5MR remained insignificant. Prenatal screening, as a health care behavior during the mother’s pregnancy, is a relatively indirect effect on children, which could explain their lack of a statistically significant effect on U5MR [16]. At the same time, there are also problems such as unqualified diagnostic services in prenatal screening in some areas (e.g., delayed prenatal diagnosis [52], biased diagnostic results by physicians [53, 54], etc.), and further standardization of services is still needed. However, the effect of a one-year lag in postpartum visits on U5MR was significant and it could reduce U5MR. The potential of timely, quality postnatal visits in reducing U5MR is well documented [55, 56]. In addition to neonatal diseases, the common causes of U5MR are important infections like lower respiratory infections, pneumonia [57], diarrhoea, and meningitis [58], which a relatively long duration and can be effectively prevented by means of scientific feeding and care. The study [59] from China showed that all mothers, whether first or second time mothers, were unsure of their infant care skills. They expressed concerns about infant feeding, defecation, and illness, suggesting that health professionals should provide postpartum mothers with the knowledge and skills they need to care for their newborns, which can be accomplished through postpartum visits. One of the very important tasks of the post-natal visit in the National Basic Public Health Service is the newborn visit [60], which provides guidance on feeding and caring for newborns. Several lifesaving newborn behaviors can be promoted, and interventions delivered, through early postnatal care. These include an assessment of the baby and treatment or referral, and counselling on breastfeeding, thermal care, hygiene, cord care and on danger signs. These measures may prevent health problems from becoming long-term, with effects on women, their babies, and their families [61].

There are some limitations that need to be considered. First, the GBD 2017 reports estimated data. Owing to the poor availability of data in some regions or countries, there may be bias between the reported and actual values. Therefore, there may be some statistical bias in our analysis. Second, in exploring the association between maternal health service utilization and U5MR, we included a limited number of control variables. We are unable to include all potential confounders due to constraints imposed by the availability of province-level data. It is possible that some variables were not measured, which may have biased the results.

## Conclusion

The decline in U5MR in China was associated with hospital delivery rate and postpartum visits rate. Hospital delivery can reduce U5MR by reducing neonatal mortality. postnatal visits have a long-term impact on reducing U5MR. The design and implementation of maternal health services may provide references to other low-income and middle-income countries.

### Electronic supplementary material

Below is the link to the electronic supplementary material.

Supplementary Material 1

## Data Availability

All the data used in the article is publicly available and accessible in the following websites: “https://www.healthdata.org/” “https://www.stats.gov.cn/sj/ndsj/” “http://www.nhc.gov.cn/mohwsbwstjxxzx/tjzxtjsj/tjsj_list.shtml” “https://www.undp.org/china/publications/national-human-development-report-special-edition”.

## Abbreviations

UN: United Nations
U5MR: under-five mortality rate
SDG: Sustainable and Development Goals
GBD: Global Burden of Disease
APC: annual percentage change
AAPC: average annual percentage change
