# Supplementary material for: Association between maternal health service utilization and under-five mortality rate in China and its provinces, 1990–2017
Source: BMC Pregnancy Childbirth. 2024 Apr 26;24:326. doi: 10.1186/s12884-024-06437-8 (PMC11055253; doi:10.1186/s12884-024-06437-8)

# supplementary file1

**Data on provinces used in the manuscript**

| **region** | **U5MR (per 1000)** | **year** | **prenatal screening rate (%)** | **hospital delivery rate (%)** | **postpartum visits rate (%)** | **Years of education per capita (years)** | **GDP per capita (Yuan)** | **Birth rate (%)** | **Disposable income per capita (Yuan)** | **Total number of health workers** | **Emissions of particulate matter in exhaust gases (million tons)** |
| --- | --- | --- | --- | --- | --- | --- | --- | --- | --- | --- | --- |
| Beijing | 5 | 2017 | 98.7 | 100 | 96.7 | 12.66511294 | 128994 | 9.06 | 57229.8 | 315238 | 3.24 |
| Tianjin | 8 | 2017 | 99.1 | 100 | 97.5 | 11.00988305 | 118944 | 7.65 | 37022.3 | 129554 | 3.75 |
| Hebei | 13 | 2017 | 96.1 | 100 | 92.1 | 9.085842141 | 45387 | 13.2 | 21484.1 | 590569 | 66.61 |
| Shanxi | 12 | 2017 | 95.6 | 100 | 91.9 | 9.862093056 | 42060 | 11.06 | 20420 | 318990 | 96.09 |
| Inner Mongolia | 13 | 2017 | 97.2 | 100 | 95.3 | 9.524502297 | 63764 | 9.47 | 26212.2 | 233062 | 161.12 |
| Liaoning | 7 | 2017 | 98 | 100 | 95.5 | 9.927868188 | 53527 | 6.49 | 27835.4 | 380915 | 109.88 |
| Jilin | 9 | 2017 | 97.4 | 100 | 95.7 | 9.506229508 | 54838 | 6.76 | 21368.3 | 224342 | 53.24 |
| Heilongjiang | 12 | 2017 | 97.6 | 100 | 95.7 | 9.363218771 | 41916 | 6.22 | 21205.8 | 300301 | 96.08 |
| Shanghai | 7 | 2017 | 98.3 | 100 | 98 | 11.40609756 | 126634 | 8.1 | 58988 | 227750 | 2.65 |
| Jiangsu | 7 | 2017 | 99.9 | 100 | 100 | 9.439000048 | 107150 | 9.71 | 35024.1 | 692473 | 66.35 |
| Zhejiang | 7 | 2017 | 98.8 | 100 | 98.1 | 9.127498465 | 92057 | 11.92 | 42045.7 | 555716 | 35.55 |
| Anhui | 12 | 2017 | 92.1 | 100 | 90 | 8.557741786 | 43401 | 14.07 | 21863.3 | 407457 | 71.12 |
| Fujian | 7 | 2017 | 97.2 | 100 | 94.3 | 9.082579765 | 82677 | 15 | 30047.7 | 300571 | 41.77 |
| Jiangxi | 16 | 2017 | 96.3 | 100 | 95.3 | 8.716377858 | 43424 | 13.79 | 22031.4 | 317798 | 72.09 |
| Shandong | 10 | 2017 | 94.9 | 100 | 93.3 | 9.061354938 | 72807 | 17.54 | 26929.9 | 917894 | 64.14 |
| Henan | 13 | 2017 | 93.4 | 100 | 89.9 | 8.894225342 | 46674 | 12.95 | 20170 | 827645 | 37.99 |
| Hubei | 12 | 2017 | 96.9 | 100 | 94.7 | 9.345668666 | 60199 | 12.6 | 23757.2 | 510044 | 48.34 |
| Hunan | 9 | 2017 | 97.2 | 99.9 | 95.9 | 9.395750611 | 49558 | 13.27 | 23102.7 | 536677 | 59.76 |
| Guangdong | 7 | 2017 | 97.5 | 100 | 95.2 | 9.696874557 | 80932 | 13.68 | 33003.3 | 864114 | 65.9 |
| Guangxi | 12 | 2017 | 98.9 | 100 | 90.2 | 8.714449385 | 38102 | 15.14 | 19904.8 | 404763 | 48.87 |
| Hainan | 16 | 2017 | 96.4 | 99.9 | 89.7 | 9.415845824 | 48430 | 14.73 | 22553.2 | 77417 | 3.17 |
| Chongqing | 13 | 2017 | 97.4 | 99.7 | 92.9 | 9.136028016 | 63442 | 11.18 | 24153 | 255854 | 24.64 |
| Sichuan | 15 | 2017 | 96.9 | 99.3 | 95.7 | 8.497601639 | 44651 | 11.26 | 20579.8 | 709899 | 65.2 |
| Guizhou | 18 | 2017 | 93.6 | 99.5 | 91.3 | 8.093306664 | 37956 | 13.98 | 16703.6 | 301876 | 68.49 |
| Yunnan | 17 | 2017 | 98.5 | 99.7 | 97.2 | 8.126672687 | 34221 | 13.53 | 18348.3 | 369151 | 67.62 |
| Tibet | 36 | 2017 | 89.5 | 92.5 | 83.4 | 5.576953748 | 39267 | 16 | 15457.3 | 33413 | 8.88 |
| Shaanxi | 15 | 2017 | 97.7 | 100 | 96.4 | 9.242388242 | 57266 | 11.11 | 20635.2 | 393846 | 51.32 |
| Gansu | 20 | 2017 | 97.3 | 99.8 | 96 | 8.597191206 | 28497 | 12.54 | 16011 | 199155 | 64.94 |
| Qinghai | 21 | 2017 | 95.8 | 97.6 | 94.8 | 7.967434648 | 44047 | 14.42 | 19001 | 56098 | 16.64 |
| Ningxia | 16 | 2017 | 99.4 | 100 | 98.5 | 9.127998465 | 50765 | 13.44 | 20561.7 | 62022 | 29.77 |
| Xinjiang | 28 | 2017 | 94.7 | 99 | 92.2 | 9.457964602 | 44941 | 15.88 | 19975.1 | 224368 | 70.4 |
| Beijing | 6 | 2016 | 98.7 | 100 | 96.5 | 12.38909476 | 118198 | 9.32 | 52530.4 | 299460 | 3.45 |
| Tianjin | 8 | 2016 | 97.2 | 100 | 96.4 | 10.69058947 | 115053 | 7.37 | 34074.5 | 122558 | 7.81 |
| Hebei | 14 | 2016 | 96 | 100 | 92.3 | 8.920444337 | 43062 | 12.42 | 19725.4 | 555115 | 125.68 |
| Shanxi | 13 | 2016 | 96 | 99.9 | 92.6 | 9.638303731 | 35532 | 10.29 | 19048.9 | 311250 | 68.15 |
| Inner Mongolia | 14 | 2016 | 97.2 | 100 | 95.4 | 9.594184703 | 72064 | 9.03 | 24126.6 | 221090 | 59.9 |
| Liaoning | 7 | 2016 | 97.7 | 100 | 94 | 9.917205066 | 50791 | 6.6 | 26039.7 | 365729 | 64.91 |
| Jilin | 9 | 2016 | 97.3 | 100 | 96 | 9.465187557 | 53868 | 5.55 | 19967 | 223250 | 21.87 |
| Heilongjiang | 13 | 2016 | 97.9 | 100 | 96.4 | 9.319133083 | 40432 | 6.12 | 19838.5 | 292297 | 44.71 |
| Shanghai | 7 | 2016 | 98.4 | 100 | 98.1 | 11.00959842 | 116562 | 9 | 54305.3 | 217061 | 7.95 |
| Jiangsu | 7 | 2016 | 100 | 100 | 100 | 9.440503233 | 96887 | 9.76 | 32070.1 | 654117 | 47.17 |
| Zhejiang | 8 | 2016 | 98.8 | 100 | 98.1 | 9.056359724 | 84916 | 11.22 | 38529 | 523598 | 18.23 |
| Anhui | 13 | 2016 | 94.1 | 100 | 92 | 8.517775979 | 39561 | 13.02 | 19998.1 | 388224 | 32.13 |
| Fujian | 8 | 2016 | 97.2 | 100 | 94.2 | 8.674952843 | 74707 | 14.5 | 27607.9 | 288205 | 23.79 |
| Jiangxi | 17 | 2016 | 96.1 | 100 | 94.9 | 8.704469684 | 40400 | 13.45 | 20109.6 | 301651 | 33.31 |
| Shandong | 10 | 2016 | 95.3 | 100 | 93.2 | 8.972128138 | 68733 | 17.89 | 24685.3 | 874110 | 87.38 |
| Henan | 13 | 2016 | 94.4 | 100 | 90.2 | 8.765971007 | 42575 | 13.26 | 18443.1 | 796480 | 42.89 |
| Hubei | 14 | 2016 | 97.2 | 100 | 95.3 | 9.244261369 | 55665 | 12.04 | 21786.6 | 494077 | 27.58 |
| Hunan | 10 | 2016 | 96.8 | 100 | 95.2 | 9.298969849 | 46382 | 13.57 | 21114.8 | 515472 | 26.21 |
| Guangdong | 8 | 2016 | 97.2 | 99.9 | 95.2 | 9.550054011 | 74016 | 11.85 | 30295.8 | 819106 | 28.17 |
| Guangxi | 13 | 2016 | 99.4 | 100 | 98.7 | 8.718630048 | 38027 | 13.82 | 18305.1 | 390601 | 26.19 |
| Hainan | 17 | 2016 | 96.4 | 99.8 | 89.9 | 9.066789825 | 44347 | 14.57 | 20653.4 | 74585 | 2.08 |
| Chongqing | 14 | 2016 | 97 | 99.6 | 93.4 | 9.00531407 | 58502 | 11.77 | 22034.1 | 242826 | 9.58 |
| Sichuan | 17 | 2016 | 96.1 | 98.7 | 95.2 | 8.254631545 | 40003 | 10.48 | 18808.3 | 670444 | 27.27 |
| Guizhou | 20 | 2016 | 94.8 | 99 | 93.4 | 7.730163404 | 33246 | 13.43 | 15121.1 | 277380 | 20.43 |
| Yunnan | 18 | 2016 | 98.5 | 99.6 | 97.8 | 7.954427295 | 31093 | 13.16 | 16719.9 | 329760 | 24.76 |
| Tibet | 38 | 2016 | 90.2 | 91.7 | 87.4 | 5.0625 | 35184 | 15.79 | 13639.2 | 29187 | 1.65 |
| Shaanxi | 16 | 2016 | 98.2 | 100 | 97.4 | 9.207568002 | 51015 | 10.64 | 18873.7 | 372646 | 28.74 |
| Gansu | 22 | 2016 | 97.2 | 99.5 | 96.2 | 8.398434825 | 27643 | 12.18 | 14670.3 | 186756 | 18.03 |
| Qinghai | 23 | 2016 | 94.7 | 97.3 | 93.5 | 7.746343593 | 43531 | 14.7 | 17301.8 | 49653 | 14.86 |
| Ningxia | 17 | 2016 | 99.2 | 99.9 | 98.5 | 9.080422265 | 47194 | 13.69 | 18832.3 | 56218 | 20.12 |
| Xinjiang | 30 | 2016 | 94.5 | 98.8 | 92.5 | 9.029413391 | 40564 | 15.34 | 18354.7 | 220039 | 45.67 |
| Beijing | 6 | 2015 | 98.6 | 100 | 96.2 | 12.14643293 | 106497 | 7.96 | 48458 | 289204 | 4.94 |
| Tianjin | 9 | 2015 | 99 | 100 | 94 | 10.49582377 | 107960 | 5.84 | 31291.4 | 118111 | 10.07 |
| Hebei | 14 | 2015 | 96 | 100 | 93.4 | 8.988104428 | 40255 | 11.35 | 18118.1 | 533286 | 157.54 |
| Shanxi | 13 | 2015 | 95.8 | 99.9 | 92.4 | 9.56493074 | 34919 | 9.98 | 17853.7 | 294851 | 144.89 |
| Inner Mongolia | 15 | 2015 | 97.1 | 99.9 | 95.5 | 9.28925436 | 71101 | 7.72 | 22310.1 | 212499 | 87.88 |
| Liaoning | 8 | 2015 | 97.7 | 100 | 94.7 | 9.776746844 | 65354 | 6.17 | 24575.6 | 348525 | 100 |
| Jilin | 10 | 2015 | 96.5 | 100 | 95.3 | 9.340247869 | 51086 | 5.87 | 18683.7 | 214193 | 44.73 |
| Heilongjiang | 14 | 2015 | 97.7 | 100 | 96.3 | 9.318429731 | 39462 | 6 | 18592.7 | 285914 | 64.41 |
| Shanghai | 7 | 2015 | 98.4 | 100 | 98.1 | 10.91565636 | 103796 | 7.52 | 49867.2 | 208444 | 12.07 |
| Jiangsu | 8 | 2015 | 100 | 100 | 100 | 9.441929741 | 87995 | 9.05 | 29538.9 | 618945 | 65.45 |
| Zhejiang | 9 | 2015 | 98.6 | 100 | 98.1 | 8.913815503 | 77644 | 10.52 | 35537.1 | 491008 | 33.02 |
| Anhui | 14 | 2015 | 93 | 100 | 90.1 | 8.740877019 | 35997 | 12.92 | 18362.6 | 377387 | 54.59 |
| Fujian | 8 | 2015 | 97 | 100 | 94.1 | 8.823247576 | 67966 | 13.9 | 25404.4 | 281330 | 34.17 |
| Jiangxi | 18 | 2015 | 95.7 | 100 | 94.4 | 8.821676046 | 36724 | 13.2 | 18437.1 | 291574 | 48.06 |
| Shandong | 11 | 2015 | 95.4 | 99.9 | 93.2 | 8.979789701 | 64168 | 12.55 | 22703.2 | 855706 | 108.25 |
| Henan | 14 | 2015 | 94.9 | 100 | 90.1 | 8.782216986 | 39123 | 12.7 | 17124.8 | 771088 | 84.61 |
| Hubei | 14 | 2015 | 96.8 | 100 | 95.4 | 9.282187207 | 50654 | 10.74 | 20025.6 | 475747 | 44.7 |
| Hunan | 10 | 2015 | 96.1 | 99.9 | 94.4 | 9.248789771 | 42754 | 13.58 | 19317.5 | 494175 | 45.45 |
| Guangdong | 8 | 2015 | 97.2 | 99.9 | 95.7 | 9.437733086 | 67503 | 11.12 | 27858.9 | 768482 | 34.78 |
| Guangxi | 14 | 2015 | 99.3 | 99.9 | 98.8 | 8.639726815 | 35190 | 14.05 | 16873.4 | 374817 | 35.59 |
| Hainan | 18 | 2015 | 96 | 99.9 | 89.3 | 9.142676699 | 40818 | 14.57 | 18979 | 71288 | 2.04 |
| Chongqing | 15 | 2015 | 96.9 | 99.5 | 93.4 | 8.883712136 | 52321 | 11.05 | 20110.1 | 227095 | 20.91 |
| Sichuan | 18 | 2015 | 96 | 98.1 | 95.2 | 8.395484698 | 36775 | 10.3 | 17221 | 646542 | 41.26 |
| Guizhou | 21 | 2015 | 94.9 | 98.8 | 93.7 | 7.74061475 | 29847 | 13 | 13696.6 | 259144 | 28.56 |
| Yunnan | 19 | 2015 | 98.3 | 99.3 | 97.7 | 8.009540533 | 28806 | 12.88 | 15222.6 | 304551 | 31.26 |
| Tibet | 41 | 2015 | 87.3 | 90.5 | 84.1 | 5.300056647 | 31999 | 15.75 | 12254.3 | 29094 | 1.71 |
| Shaanxi | 17 | 2015 | 98.3 | 100 | 97.4 | 9.513343142 | 47626 | 10.1 | 17395 | 349892 | 60.36 |
| Gansu | 24 | 2015 | 97.4 | 99.4 | 96.7 | 8.38881923 | 26165 | 12.36 | 13466.6 | 181445 | 29.54 |
| Qinghai | 25 | 2015 | 95.2 | 97.2 | 93.4 | 7.473942374 | 41252 | 14.72 | 15812.7 | 48440 | 24.6 |
| Ningxia | 18 | 2015 | 99.3 | 99.9 | 98.7 | 8.821183232 | 43805 | 12.62 | 17329.1 | 52568 | 22.99 |
| Xinjiang | 32 | 2015 | 94.2 | 98.6 | 92.3 | 9.027499242 | 40036 | 15.59 | 16859.1 | 208536 | 59.59 |
| Beijing | 6 | 2014 | 98.9 | 100 | 96.6 | 11.47266461 | 99995 | 9.75 | 44488.6 | 274036 | 5.74 |
| Tianjin | 9 | 2014 | 95.8 | 100 | 94.9 | 10.27591119 | 105231 | 8.19 | 28832.3 | 111672 | 13.95 |
| Hebei | 15 | 2014 | 95.8 | 100 | 92.8 | 8.788144523 | 39984 | 13.18 | 16647.4 | 512877 | 179.77 |
| Shanxi | 14 | 2014 | 95.1 | 99.9 | 92.3 | 9.198107233 | 35070 | 10.92 | 16538.3 | 289266 | 150.68 |
| Inner Mongolia | 16 | 2014 | 97.1 | 99.9 | 96.2 | 8.886247182 | 71046 | 9.31 | 20559.3 | 202999 | 102.15 |
| Liaoning | 8 | 2014 | 98.2 | 100 | 96.1 | 9.737310698 | 65201 | 6.49 | 22820.2 | 339187 | 112.07 |
| Jilin | 10 | 2014 | 95.8 | 100 | 94.5 | 9.253306909 | 50160 | 6.62 | 17520.4 | 206092 | 47.51 |
| Heilongjiang | 14 | 2014 | 97.5 | 100 | 96.3 | 9.230693102 | 39226 | 7.37 | 17404.4 | 283494 | 79.35 |
| Shanghai | 8 | 2014 | 98.6 | 100 | 98.4 | 10.54434975 | 97370 | 8.35 | 45965.8 | 201735 | 14.17 |
| Jiangsu | 8 | 2014 | 100 | 100 | 100 | 9.20702586 | 81874 | 9.45 | 27172.8 | 589559 | 76.37 |
| Zhejiang | 9 | 2014 | 98.7 | 100 | 98.2 | 8.906471816 | 73002 | 10.51 | 32657.6 | 455809 | 37.97 |
| Anhui | 14 | 2014 | 90.7 | 99.9 | 84.9 | 8.62336626 | 34425 | 12.86 | 16795.5 | 365662 | 65.28 |
| Fujian | 9 | 2014 | 96.7 | 100 | 93.6 | 8.675740426 | 63472 | 13.7 | 23330.9 | 273602 | 36.79 |
| Jiangxi | 19 | 2014 | 94.9 | 99.9 | 93.6 | 8.793756546 | 34674 | 13.24 | 16734.2 | 280766 | 46.23 |
| Shandong | 11 | 2014 | 94.6 | 100 | 93.1 | 8.884172185 | 60879 | 14.23 | 20864.2 | 838474 | 120.81 |
| Henan | 15 | 2014 | 94.2 | 100 | 87.7 | 8.896255423 | 37072 | 12.8 | 15695.2 | 745144 | 88.21 |
| Hubei | 14 | 2014 | 96.8 | 100 | 95.6 | 8.996642868 | 47145 | 11.86 | 18283.2 | 438164 | 50.4 |
| Hunan | 11 | 2014 | 95.9 | 99.9 | 94 | 8.928041586 | 40271 | 13.52 | 17621.7 | 462694 | 49.62 |
| Guangdong | 8 | 2014 | 97 | 99.8 | 95.3 | 9.188860037 | 63469 | 10.8 | 25685 | 732573 | 44.95 |
| Guangxi | 14 | 2014 | 99.3 | 99.9 | 98.9 | 8.669368182 | 33090 | 14.07 | 15557.1 | 357622 | 40.29 |
| Hainan | 19 | 2014 | 95.8 | 99.9 | 89.1 | 9.017140346 | 38924 | 14.56 | 17476.5 | 66556 | 2.32 |
| Chongqing | 16 | 2014 | 96.5 | 98.9 | 93.4 | 8.826935477 | 47850 | 10.67 | 18351.9 | 210501 | 22.61 |
| Sichuan | 19 | 2014 | 95.8 | 97.5 | 94.9 | 8.256990869 | 35128 | 10.22 | 15749 | 626938 | 42.86 |
| Guizhou | 22 | 2014 | 95.8 | 98.5 | 94.5 | 7.988277728 | 26437 | 12.98 | 12371.1 | 237518 | 37.79 |
| Yunnan | 21 | 2014 | 98.5 | 98.9 | 97.8 | 7.718519341 | 27264 | 12.65 | 13772.2 | 282760 | 36.68 |
| Tibet | 42 | 2014 | 88.9 | 85 | 75.6 | 4.195902689 | 29252 | 15.76 | 10730.2 | 26531 | 1.39 |
| Shaanxi | 17 | 2014 | 98.1 | 99.9 | 97 | 9.029145211 | 46929 | 10.13 | 15836.7 | 336288 | 70.91 |
| Gansu | 25 | 2014 | 97.9 | 99.3 | 96.8 | 8.21802442 | 26433 | 12.21 | 12184.7 | 178968 | 34.58 |
| Qinghai | 26 | 2014 | 95.7 | 96.8 | 94.3 | 7.913161465 | 39671 | 14.67 | 14374 | 46363 | 23.99 |
| Ningxia | 19 | 2014 | 99.3 | 99.9 | 98.7 | 8.441722639 | 41834 | 13.1 | 15906.8 | 50714 | 23.92 |
| Xinjiang | 33 | 2014 | 94.3 | 98.7 | 91.7 | 9.047591173 | 40648 | 16.44 | 15096.6 | 199649 | 81.39 |
| Beijing | 6 | 2013 | 98.8 | 100 | 96.2 | 11.61628117 | 94648 | 8.93 | 40830 | 263146 | 5.93 |
| Tianjin | 9 | 2013 | 95.5 | 100 | 94.9 | 10.30806424 | 100105 | 8.28 | 26359.2 | 106527 | 8.75 |
| Hebei | 15 | 2013 | 95.9 | 99.9 | 92.5 | 8.824378681 | 38909 | 13.04 | 15189.6 | 492012 | 131.33 |
| Shanxi | 14 | 2013 | 90.6 | 99.9 | 90.6 | 9.249466496 | 34984 | 10.81 | 15119.7 | 283860 | 102.67 |
| Inner Mongolia | 16 | 2013 | 97.3 | 99.7 | 96 | 8.909398554 | 67836 | 8.98 | 18692.9 | 195952 | 82.21 |
| Liaoning | 8 | 2013 | 96.5 | 100 | 95.6 | 9.906657122 | 61996 | 6.09 | 20817.8 | 338443 | 67.06 |
| Jilin | 10 | 2013 | 96.2 | 100 | 94.9 | 9.287669339 | 47428 | 5.36 | 15998.1 | 200184 | 32.02 |
| Heilongjiang | 15 | 2013 | 97.8 | 100 | 96.6 | 9.358109893 | 37697 | 6.86 | 15903.4 | 279122 | 72.25 |
| Shanghai | 8 | 2013 | 98.5 | 100 | 98.2 | 10.31460674 | 90993 | 8.18 | 42173.6 | 192333 | 8.09 |
| Jiangsu | 8 | 2013 | 100 | 100 | 100 | 9.285046729 | 75354 | 9.44 | 24775.5 | 551113 | 50 |
| Zhejiang | 9 | 2013 | 98.6 | 100 | 98.2 | 9.194213533 | 68805 | 10.01 | 29775 | 427072 | 31.97 |
| Anhui | 15 | 2013 | 89.8 | 99.9 | 82.9 | 8.43294483 | 32001 | 12.88 | 15154.3 | 353799 | -41.86 |
| Fujian | 9 | 2013 | 96.5 | 100 | 93.7 | 8.558159783 | 58145 | 12.2 | 21217.9 | 261784 | 25.94 |
| Jiangxi | 20 | 2013 | 94.5 | 99.8 | 94 | 9.14459829 | 31930 | 13.19 | 15099.7 | 269819 | 35.63 |
| Shandong | 12 | 2013 | 95.5 | 100 | 94.2 | 8.825825227 | 56885 | 11.41 | 19008.3 | 819348 | 69.67 |
| Henan | 15 | 2013 | 93.3 | 99.9 | 86.3 | 8.702407556 | 34211 | 12.27 | 14203.7 | 716306 | 64.13 |
| Hubei | 15 | 2013 | 95.9 | 100 | 94.8 | 9.225593312 | 42826 | 11.08 | 16472.5 | 411184 | 35.95 |
| Hunan | 11 | 2013 | 96 | 99.9 | 93.9 | 8.872620189 | 36943 | 13.5 | 16004.9 | 442224 | 35.87 |
| Guangdong | 9 | 2013 | 96.9 | 99.5 | 95.2 | 9.144569869 | 58833 | 10.71 | 23420.7 | 708036 | 35.4 |
| Guangxi | 15 | 2013 | 96.3 | 99.8 | 98.6 | 8.516344474 | 30741 | 14.28 | 14082.3 | 334849 | 28.95 |
| Hainan | 19 | 2013 | 94.4 | 99.8 | 87 | 9.099925981 | 35663 | 14.59 | 15733.3 | 63468 | 1.8 |
| Chongqing | 16 | 2013 | 95.1 | 98.5 | 91.2 | 8.582377138 | 43223 | 10.37 | 16568.7 | 197667 | 19.12 |
| Sichuan | 19 | 2013 | 95.6 | 97 | 94.3 | 8.342095597 | 32617 | 9.9 | 14231 | 596001 | 29.6 |
| Guizhou | 23 | 2013 | 96 | 98.1 | 94.8 | 7.951742023 | 23151 | 13.05 | 11083.1 | 221575 | 30.13 |
| Yunnan | 21 | 2013 | 97.6 | 98.2 | 97.9 | 7.764596901 | 25322 | 12.6 | 12577.9 | 265531 | 38.69 |
| Tibet | 43 | 2013 | 66.6 | 82.3 | 68.5 | 4.343478261 | 26326 | 15.77 | 9740.4 | 24653 | 0.68 |
| Shaanxi | 18 | 2013 | 96.6 | 99.9 | 97.4 | 9.163113449 | 43117 | 10.01 | 14371.5 | 321908 | 53.77 |
| Gansu | 26 | 2013 | 96.6 | 99.1 | 95.7 | 8.256243378 | 24539 | 12.16 | 10954.4 | 160695 | 22.66 |
| Qinghai | 26 | 2013 | 89.4 | 95.5 | 88.6 | 7.838335607 | 36875 | 14.16 | 12947.8 | 44685 | 17.38 |
| Ningxia | 20 | 2013 | 99.2 | 99.8 | 98.4 | 8.594888308 | 39613 | 13.12 | 14565.8 | 47609 | 23.06 |
| Xinjiang | 34 | 2013 | 94.8 | 98.8 | 92.4 | 8.85902589 | 37553 | 15.84 | 13669.6 | 189578 | 75.59 |
| Beijing | 6 | 2012 | 99 | 100 | 97.1 | 11.46281997 | 87475 | 9.05 | 36468.75 | 253164 | 6.68 |
| Tianjin | 9 | 2012 | 95.9 | 100 | 94.7 | 10.28322148 | 93173 | 8.75 | 29626.41 | 104201 | 8.41 |
| Hebei | 16 | 2012 | 95.7 | 99.8 | 93.1 | 8.651994843 | 36584 | 12.88 | 20543.44 | 463283 | 123.59 |
| Shanxi | 15 | 2012 | 92.9 | 99.8 | 90.5 | 9.286177258 | 33628 | 10.7 | 20411.71 | 279466 | 107.09 |
| Inner Mongolia | 17 | 2012 | 97.2 | 99.8 | 95.6 | 9.109756098 | 63886 | 9.17 | 23150.26 | 183875 | 83.3 |
| Liaoning | 8 | 2012 | 98.6 | 100 | 97 | 9.713008684 | 56649 | 6.15 | 23222.67 | 329679 | 72.63 |
| Jilin | 10 | 2012 | 92.9 | 100 | 91.8 | 9.165007569 | 43415 | 5.73 | 20208.04 | 196395 | 26.48 |
| Heilongjiang | 15 | 2012 | 97.7 | 100 | 96.3 | 9.108501045 | 35711 | 7.3 | 17759.75 | 270687 | 69.93 |
| Shanghai | 8 | 2012 | 78.2 | 100 | 78 | 10.42355784 | 85373 | 9.56 | 40188.34 | 183416 | 8.71 |
| Jiangsu | 8 | 2012 | 100 | 100 | 100 | 9.126835931 | 68347 | 9.44 | 29676.97 | 519709 | 44.32 |
| Zhejiang | 10 | 2012 | 98.6 | 100 | 98.2 | 9.061684186 | 63374 | 10.12 | 34550.3 | 400094 | 25.4 |
| Anhui | 16 | 2012 | 84.4 | 99.7 | 73.6 | 8.413801342 | 28792 | 13 | 21024.21 | 334842 | 46.21 |
| Fujian | 10 | 2012 | 96.2 | 100 | 93.1 | 8.485672808 | 52763 | 12.74 | 28055.24 | 236756 | 25.26 |
| Jiangxi | 21 | 2012 | 94.8 | 99.7 | 94.1 | 8.783655526 | 28800 | 13.46 | 19860.36 | 259552 | 35.74 |
| Shandong | 12 | 2012 | 96.1 | 100 | 95.3 | 8.681790459 | 51768 | 11.9 | 25755.19 | 738868 | 69.53 |
| Henan | 16 | 2012 | 92.5 | 99.5 | 85.1 | 8.596527026 | 31499 | 11.87 | 20442.62 | 652564 | 59.98 |
| Hubei | 15 | 2012 | 97.7 | 99.9 | 96.5 | 9.079341151 | 38572 | 11 | 20839.59 | 386415 | 34.97 |
| Hunan | 12 | 2012 | 95.2 | 99.8 | 93.1 | 8.648036165 | 33480 | 13.58 | 21318.76 | 403546 | 34.07 |
| Guangdong | 9 | 2012 | 95.7 | 99.1 | 94.2 | 9.250206742 | 54095 | 11.6 | 30226.71 | 662462 | 32.83 |
| Guangxi | 15 | 2012 | 95.7 | 99.7 | 98.3 | 8.359073469 | 27952 | 14.2 | 21242.8 | 303759 | 29.97 |
| Hainan | 20 | 2012 | 94.1 | 99.6 | 88.1 | 9.044736454 | 32377 | 14.66 | 20917.71 | 59285 | 1.66 |
| Chongqing | 17 | 2012 | 95.2 | 97.6 | 91 | 8.535858389 | 38914 | 10.86 | 22968.14 | 184055 | 18.23 |
| Sichuan | 20 | 2012 | 93.3 | 96.2 | 92.3 | 8.379161187 | 29608 | 9.89 | 20306.99 | 549023 | 29.58 |
| Guizhou | 25 | 2012 | 96.2 | 97.8 | 94.9 | 7.565207598 | 19710 | 13.27 | 18700.51 | 191079 | 29.45 |
| Yunnan | 23 | 2012 | 96.7 | 96.5 | 97.1 | 6.766491461 | 22195 | 12.63 | 21074.5 | 233361 | 39.06 |
| Tibet | 45 | 2012 | 61.6 | 73.3 | 52.7 | 5.028730703 | 22936 | 15.48 | 18028.32 | 21558 | 0.66 |
| Shaanxi | 19 | 2012 | 97.2 | 99.8 | 97.3 | 9.028469751 | 38564 | 10.12 | 20733.88 | 293775 | 46.21 |
| Gansu | 28 | 2012 | 96.8 | 98.3 | 95.6 | 8.190023871 | 21978 | 12.11 | 17156.89 | 151899 | 20.76 |
| Qinghai | 28 | 2012 | 89.3 | 94.1 | 88.5 | 7.513593113 | 33181 | 14.3 | 17566.28 | 40831 | 15.64 |
| Ningxia | 22 | 2012 | 99.5 | 99.8 | 98.7 | 8.275549284 | 36394 | 13.26 | 19831.41 | 44021 | 19.83 |
| Xinjiang | 36 | 2012 | 94.8 | 98.6 | 91.5 | 8.915552927 | 33796 | 15.32 | 17920.68 | 177085 | 69.61 |
| Beijing | 6 | 2011 | 99.1 | 100 | 97.3 | 11.21557213 | 81658 | 8.29 | 32903.03 | 235652 | 6.58 |
| Tianjin | 9 | 2011 | 97.3 | 100 | 93.6 | 10.19003539 | 85213 | 8.58 | 26920.86 | 100169 | 7.59 |
| Hebei | 17 | 2011 | 95.3 | 99.6 | 91.7 | 8.612214332 | 33969 | 13.02 | 18292.23 | 449108 | 132.25 |
| Shanxi | 15 | 2011 | 89.6 | 99.4 | 87.4 | 9.071835804 | 31357 | 10.47 | 18123.87 | 271627 | 112.99 |
| Inner Mongolia | 17 | 2011 | 95.9 | 99.7 | 93.7 | 9.100424681 | 57974 | 8.94 | 20407.57 | 175226 | 73.99 |
| Liaoning | 9 | 2011 | 91.6 | 100 | 95.8 | 9.341725617 | 50760 | 5.71 | 20466.84 | 319116 | 69.32 |
| Jilin | 11 | 2011 | 94.1 | 100 | 92.5 | 9.010195412 | 38460 | 6.53 | 17796.57 | 192940 | 43.22 |
| Heilongjiang | 16 | 2011 | 96.5 | 100 | 94.7 | 9.02062548 | 32819 | 6.99 | 15696.18 | 266066 | 65.59 |
| Shanghai | 8 | 2011 | 82.1 | 100 | 81.9 | 10.27159464 | 82560 | 6.97 | 36230.48 | 176632 | 8.98 |
| Jiangsu | 9 | 2011 | 100 | 100 | 100 | 9.042117703 | 62290 | 9.59 | 26340.73 | 481818 | 52.74 |
| Zhejiang | 10 | 2011 | 98.5 | 100 | 97.9 | 8.694770206 | 59249 | 9.47 | 30970.68 | 374157 | 32.33 |
| Anhui | 17 | 2011 | 76.3 | 99.2 | 60 | 8.179934986 | 25659 | 12.23 | 18606.13 | 315382 | 45.22 |
| Fujian | 10 | 2011 | 96.5 | 100 | 93 | 8.708419378 | 47377 | 11.41 | 24907.4 | 217586 | 22.53 |
| Jiangxi | 22 | 2011 | 94.6 | 99.5 | 91.5 | 8.664131088 | 26150 | 13.48 | 17494.87 | 244570 | 39.6 |
| Shandong | 13 | 2011 | 95 | 100 | 95.8 | 8.58317514 | 47335 | 11.5 | 22791.84 | 689628 | 78.38 |
| Henan | 16 | 2011 | 90 | 99.3 | 82.1 | 8.628008663 | 28661 | 11.56 | 18194.8 | 623494 | 66.82 |
| Hubei | 16 | 2011 | 97.6 | 99.9 | 96.2 | 8.934795341 | 34197 | 10.39 | 18373.87 | 365175 | 34.61 |
| Hunan | 12 | 2011 | 94.6 | 99.7 | 92.2 | 8.727897442 | 29880 | 13.35 | 18844.05 | 387975 | 38.44 |
| Guangdong | 9 | 2011 | 96.7 | 98.7 | 95.8 | 9.227384038 | 50807 | 10.45 | 26897.48 | 626571 | 32.43 |
| Guangxi | 16 | 2011 | 94.4 | 99.4 | 97.2 | 8.522942906 | 25326 | 13.71 | 18854.06 | 283543 | 28.83 |
| Hainan | 21 | 2011 | 91.1 | 99.6 | 76.7 | 8.802775749 | 28898 | 14.72 | 18368.95 | 56893 | 1.58 |
| Chongqing | 18 | 2011 | 95.4 | 96.7 | 91.5 | 8.664290897 | 34500 | 9.88 | 20249.7 | 170799 | 18.1 |
| Sichuan | 22 | 2011 | 91.8 | 94.7 | 90.5 | 8.133549932 | 26133 | 9.79 | 17899.12 | 505712 | 38.59 |
| Guizhou | 27 | 2011 | 94.5 | 96.3 | 93.5 | 7.506257614 | 16413 | 13.31 | 16495.01 | 169098 | 30.35 |
| Yunnan | 24 | 2011 | 96.7 | 93.8 | 95.8 | 7.616259719 | 19265 | 12.71 | 18575.62 | 215335 | 38.22 |
| Tibet | 46 | 2011 | 66.7 | 62.5 | 50.9 | 5.461146497 | 20077 | 15.39 | 16195.56 | 22234 | 1 |
| Shaanxi | 20 | 2011 | 96.3 | 99.5 | 96.1 | 8.850369581 | 33464 | 9.75 | 18245.23 | 275464 | 46.34 |
| Gansu | 29 | 2011 | 96.2 | 96.7 | 94.8 | 8.065849029 | 19595 | 12.08 | 14988.68 | 145699 | 23.62 |
| Qinghai | 29 | 2011 | 87.4 | 93.1 | 87.6 | 7.693309385 | 29522 | 14.43 | 15603.31 | 38785 | 13.83 |
| Ningxia | 23 | 2011 | 96.9 | 99.5 | 96.1 | 8.29950446 | 33043 | 13.65 | 17578.92 | 41758 | 21.55 |
| Xinjiang | 37 | 2011 | 92.6 | 98.3 | 88.2 | 9.035581395 | 30087 | 14.99 | 15513.62 | 167828 | 53.19 |
| Beijing | 7 | 2010 | 98.9 | 100 | 97.1 | 10.69425 | 75943 | 7.48 | 29072.93 | 223586 | 6.5307 |
| Tianjin | 10 | 2010 | 98.4 | 100 | 95.3 | 9.55692 | 72994 | 8.18 | 24292.6 | 96732 | 7.2522 |
| Hebei | 17 | 2010 | 94.6 | 99.3 | 91.2 | 8.09514 | 28668 | 13.22 | 16263.43 | 437415 | 82.064 |
| Shanxi | 16 | 2010 | 92.9 | 98.9 | 86.8 | 8.56875 | 26283 | 10.68 | 15647.66 | 275955 | 98.5923 |
| Inner Mongolia | 18 | 2010 | 95.5 | 99.6 | 93.3 | 8.4009 | 47347 | 9.3 | 17698.15 | 168884 | 80.7329 |
| Liaoning | 9 | 2010 | 97.9 | 100 | 96.7 | 8.93325 | 42355 | 6.68 | 17712.58 | 316828 | 80.0632 |
| Jilin | 11 | 2010 | 90.7 | 99.9 | 88.5 | 8.73717 | 31599 | 7.91 | 15411.47 | 187106 | 35.3792 |
| Heilongjiang | 16 | 2010 | 96.4 | 99.8 | 93.2 | 8.66094 | 27076 | 7.35 | 13856.51 | 262600 | 47.8719 |
| Shanghai | 9 | 2010 | 87 | 100 | 86.9 | 9.90231 | 76074 | 7.05 | 31838.08 | 171935 | 11.1846 |
| Jiangsu | 9 | 2010 | 100 | 100 | 100 | 8.49027 | 52840 | 9.73 | 22944.26 | 459025 | 48.6402 |
| Zhejiang | 11 | 2010 | 98.4 | 100 | 97.7 | 8.05737 | 51711 | 10.27 | 27359.02 | 352871 | 31.3639 |
| Anhui | 18 | 2010 | 74.4 | 98.7 | 60.3 | 7.39557 | 20888 | 12.7 | 15788.17 | 309318 | 51.9109 |
| Fujian | 11 | 2010 | 97.4 | 99.9 | 94.3 | 8.11707 | 40025 | 11.27 | 21781.31 | 199519 | 27.8789 |
| Jiangxi | 23 | 2010 | 94.1 | 99.4 | 93.2 | 7.7076 | 21253 | 13.72 | 15481.12 | 230945 | 38.7967 |
| Shandong | 13 | 2010 | 97.7 | 99.9 | 97 | 8.08506 | 41106 | 11.65 | 19945.83 | 645889 | 58.1082 |
| Henan | 17 | 2010 | 91.2 | 98.9 | 83.7 | 7.81302 | 24446 | 11.52 | 15930.26 | 591059 | 77.3484 |
| Hubei | 17 | 2010 | 97 | 99.8 | 94.3 | 8.36007 | 27906 | 10.36 | 16058.37 | 349495 | 33.957 |
| Hunan | 13 | 2010 | 94 | 99.3 | 91.6 | 8.15427 | 24719 | 13.1 | 16565.7 | 370261 | 70.6607 |
| Guangdong | 10 | 2010 | 96.8 | 97.7 | 95.8 | 8.52027 | 44736 | 11.18 | 23897.8 | 592800 | 41.5361 |
| Guangxi | 17 | 2010 | 97.8 | 98.7 | 95.4 | 7.61007 | 20219 | 14.13 | 17063.89 | 266138 | 57.9294 |
| Hainan | 22 | 2010 | 91.9 | 99.4 | 76.5 | 8.04597 | 23831 | 14.71 | 15581.05 | 51985 | 1.4682 |
| Chongqing | 19 | 2010 | 94.1 | 94.4 | 89.3 | 7.87779 | 27596 | 9.17 | 17532.43 | 160055 | 29.1303 |
| Sichuan | 23 | 2010 | 91.5 | 92.5 | 89.4 | 7.56852 | 21182 | 8.93 | 15461.16 | 467126 | 48.2594 |
| Guizhou | 29 | 2010 | 92.7 | 88.1 | 91.9 | 6.71103 | 13119 | 13.96 | 14142.74 | 154246 | 33.8429 |
| Yunnan | 26 | 2010 | 96.1 | 90.9 | 94.4 | 6.9483 | 15752 | 13.1 | 16064.54 | 207663 | 22.9978 |
| Tibet | 48 | 2010 | 64.2 | 53.6 | 52.8 | 4.70157 | 17319 | 15.8 | 14980.47 | 16694 | 0.324 |
| Shaanxi | 22 | 2010 | 97.6 | 99.4 | 96.5 | 8.49333 | 27133 | 9.73 | 15695.21 | 260056 | 34.8606 |
| Gansu | 31 | 2010 | 93.8 | 93.6 | 91.6 | 7.40985 | 16113 | 12.05 | 13188.55 | 137501 | 25.573 |
| Qinghai | 32 | 2010 | 86.1 | 91.5 | 86.3 | 6.9432 | 24115 | 14.94 | 13854.99 | 35224 | 17.4049 |
| Ningxia | 25 | 2010 | 99.2 | 98.5 | 98.3 | 7.68534 | 26860 | 14.14 | 15344.49 | 39674 | 23.2794 |
| Xinjiang | 39 | 2010 | 93.8 | 97.6 | 87.7 | 8.03823 | 25034 | 15.99 | 13643.77 | 158917 | 52.8627 |
| Beijing | 7 | 2009 | 99 | 100 | 97.2 | 10.86484798 | 70452 | 8.06 | 26738.48 | 211714 | 6.1692718 |
| Tianjin | 10 | 2009 | 98.4 | 100 | 91.5 | 9.881406436 | 62574 | 8.3 | 21402.01 | 93366 | 7.9268899 |
| Hebei | 18 | 2009 | 93.4 | 99 | 89.2 | 8.368769437 | 24581 | 12.93 | 14718.25 | 407351 | 94.5624065 |
| Shanxi | 17 | 2009 | 87.1 | 97.2 | 79.6 | 8.799790795 | 21522 | 10.87 | 13996.55 | 264352 | 107.4613305 |
| Inner Mongolia | 19 | 2009 | 94.6 | 99.5 | 92.2 | 8.415061898 | 40282 | 9.57 | 15849.19 | 177697 | 65.7875187 |
| Liaoning | 10 | 2009 | 98 | 99.9 | 96.3 | 9.119127426 | 35239 | 6.06 | 15761.38 | 310007 | 83.9589008 |
| Jilin | 12 | 2009 | 84 | 99.9 | 82.2 | 8.820572312 | 26595 | 6.69 | 14006.27 | 180775 | 44.9972612 |
| Heilongjiang | 17 | 2009 | 93.7 | 99.4 | 88.6 | 8.680243273 | 22447 | 7.48 | 12565.98 | 242973 | 53.3998532 |
| Shanghai | 9 | 2009 | 84.7 | 100 | 84.4 | 10.4103461 | 78989 | 8.64 | 28837.78 | 169506 | 11.0128312 |
| Jiangsu | 10 | 2009 | 98.9 | 100 | 95.9 | 8.468404608 | 44744 | 9.55 | 20551.72 | 437008 | 49.3825103 |
| Zhejiang | 12 | 2009 | 98.2 | 100 | 97.3 | 8.303890447 | 44641 | 10.22 | 24610.81 | 327014 | 35.7902273 |
| Anhui | 19 | 2009 | 69.5 | 97.6 | 61 | 7.574720133 | 16407.66 | 13.07 | 14085.74 | 305499 | 56.4766592 |
| Fujian | 11 | 2009 | 96.9 | 99.7 | 93.3 | 8.248413844 | 33840 | 12.2 | 19576.83 | 184866 | 26.9938484 |
| Jiangxi | 25 | 2009 | 92 | 98.4 | 90.1 | 8.454323824 | 17335 | 13.87 | 14021.54 | 223480 | 42.7589384 |
| Shandong | 14 | 2009 | 97.9 | 99.8 | 96.9 | 8.252687899 | 35894 | 11.7 | 17811.04 | 602143 | 63.7796751 |
| Henan | 18 | 2009 | 89.4 | 97.3 | 79.8 | 8.335881399 | 20597 | 11.45 | 14371.56 | 572773 | 84.6153886 |
| Hubei | 18 | 2009 | 96.3 | 99.4 | 93.7 | 8.412355663 | 22677 | 9.48 | 14367.48 | 337113 | 39.9744346 |
| Hunan | 14 | 2009 | 93.1 | 98.5 | 90.5 | 8.403961517 | 20428 | 13.05 | 15084.31 | 351160 | 91.6224009 |
| Guangdong | 11 | 2009 | 94.1 | 96 | 94.2 | 8.803020674 | 41166 | 11.78 | 21574.72 | 555799 | 40.6554474 |
| Guangxi | 19 | 2009 | 96.9 | 97.7 | 94.6 | 8.054670225 | 16045 | 14.17 | 15451.48 | 245611 | 72.6272893 |
| Hainan | 24 | 2009 | 91.8 | 98.9 | 74.3 | 8.368150685 | 19254 | 14.66 | 13750.85 | 49898 | 1.8114849 |
| Chongqing | 20 | 2009 | 88.8 | 91.4 | 83.7 | 7.879182156 | 22920 | 9.9 | 15748.67 | 146033 | 29.8323107 |
| Sichuan | 25 | 2009 | 89.8 | 89.8 | 87.5 | 7.637751744 | 17339 | 9.15 | 13839.4 | 437760 | 39.8554689 |
| Guizhou | 32 | 2009 | 91 | 77.2 | 89.2 | 7.048397415 | 10309 | 13.65 | 12862.53 | 143868 | 53.8544145 |
| Yunnan | 27 | 2009 | 95.3 | 86.3 | 93.1 | 6.874660922 | 13539 | 12.53 | 14423.93 | 196796 | 28.0098579 |
| Tibet | 51 | 2009 | 66.1 | 51.7 | 54.7 | 4.530947368 | 15295 | 15.31 | 13544.41 | 16040 | 0.3572887 |
| Shaanxi | 24 | 2009 | 96.9 | 98.8 | 95.6 | 8.493583882 | 21688 | 10.24 | 14128.76 | 245352 | 35.029344 |
| Gansu | 33 | 2009 | 92.6 | 89.1 | 89.7 | 7.243250614 | 12872 | 13.32 | 11929.78 | 126388 | 24.547378 |
| Qinghai | 33 | 2009 | 81.3 | 87 | 83.9 | 7.35957027 | 19454 | 14.51 | 12691.85 | 34429 | 14.4992123 |
| Ningxia | 27 | 2009 | 98.9 | 96.3 | 98 | 8.132992327 | 21777 | 14.38 | 14024.7 | 37734 | 13.4188471 |
| Xinjiang | 41 | 2009 | 92.3 | 96.4 | 84.5 | 8.562951496 | 19942 | 15.99 | 12257.52 | 146943 | 50.1314838 |
| Beijing | 7 | 2008 | 98.9 | 100 | 96.9 | 10.68837308 | 63029 | 8.17 | 24724.89 | 194307 | 6.3581 |
| Tianjin | 11 | 2008 | 80.5 | 100 | 76 | 9.722966014 | 55473 | 8.13 | 19422.53 | 85886 | 7.7641 |
| Hebei | 20 | 2008 | 93.1 | 97.6 | 89.4 | 8.309838247 | 23239 | 13.04 | 13441.09 | 303232 | 107.559 |
| Shanxi | 18 | 2008 | 84.4 | 94.6 | 77.2 | 8.738591782 | 20398 | 11.32 | 13119.05 | 191152 | 120.2859 |
| Inner Mongolia | 20 | 2008 | 93.5 | 98.5 | 90.4 | 8.295053515 | 32214 | 9.81 | 14432.55 | 131175 | 78.8442 |
| Liaoning | 11 | 2008 | 98 | 99.8 | 96.3 | 8.96832929 | 31259 | 6.32 | 14392.69 | 274890 | 95.9464 |
| Jilin | 12 | 2008 | 80.5 | 99.4 | 79.1 | 8.814510003 | 23514 | 6.65 | 12829.45 | 162303 | 44.2328 |
| Heilongjiang | 18 | 2008 | 91.2 | 98.7 | 83.8 | 8.641154821 | 21727 | 7.91 | 11581.28 | 203528 | 58.832 |
| Shanghai | 9 | 2008 | 80.6 | 100 | 80.3 | 10.31880425 | 73124 | 8.89 | 26674.9 | 162160 | 11.4268 |
| Jiangsu | 11 | 2008 | 98.1 | 100 | 93.2 | 8.372519433 | 39622 | 9.34 | 18679.52 | 360845 | 54.0029 |
| Zhejiang | 13 | 2008 | 97.9 | 99.9 | 97 | 8.142886683 | 42214 | 10.2 | 22726.66 | 288340 | 34.592 |
| Anhui | 20 | 2008 | 67.6 | 96.7 | 57.1 | 7.400198262 | 14485 | 13.05 | 12990.35 | 227438 | 61.3344 |
| Fujian | 12 | 2008 | 96.2 | 99.5 | 92.4 | 7.745241872 | 30123 | 12.2 | 17961.45 | 124213 | 28.8156 |
| Jiangxi | 27 | 2008 | 90.2 | 96.9 | 88.9 | 8.191528239 | 14781 | 13.92 | 12866.44 | 168472 | 48.3626 |
| Shandong | 15 | 2008 | 98 | 99.6 | 96.6 | 8.220371019 | 33083 | 11.25 | 16305.41 | 438009 | 70.5359 |
| Henan | 20 | 2008 | 88.9 | 95.8 | 79.4 | 8.288657036 | 19593 | 11.42 | 13231.11 | 396078 | 90.0259 |
| Hubei | 19 | 2008 | 95.3 | 98.7 | 93.5 | 8.404753661 | 19860 | 9.21 | 13152.86 | 284832 | 44.6125 |
| Hunan | 16 | 2008 | 92.7 | 96.9 | 89.1 | 8.367682149 | 17521 | 12.68 | 13821.16 | 281421 | 93.2443 |
| Guangdong | 11 | 2008 | 92 | 93.8 | 91.7 | 8.702569832 | 37589 | 11.8 | 19732.86 | 479817 | 52.6682 |
| Guangxi | 21 | 2008 | 95.2 | 95.7 | 91.5 | 7.950234222 | 14966 | 14.4 | 14146.04 | 190152 | 66.2197 |
| Hainan | 25 | 2008 | 87.8 | 97.4 | 70.3 | 8.287558519 | 17175 | 14.71 | 12607.84 | 42682 | 1.8445 |
| Chongqing | 22 | 2008 | 87.6 | 88.6 | 82.7 | 7.744523115 | 18025 | 10.1 | 14367.55 | 109014 | 33.7616 |
| Sichuan | 31 | 2008 | 88.6 | 85.7 | 85.2 | 7.47118071 | 15378 | 9.54 | 12633.38 | 324525 | 46.5978 |
| Guizhou | 35 | 2008 | 90.6 | 68.9 | 88 | 7.010502341 | 8824 | 13.49 | 11758.76 | 106038 | 45.5115 |
| Yunnan | 29 | 2008 | 94.9 | 80.1 | 91.7 | 6.866766585 | 12587 | 12.63 | 13250.22 | 151859 | 32.6696 |
| Tibet | 54 | 2008 | 67.3 | 44 | 52.5 | 4.694339623 | 13861 | 15.5 | 12481.51 | 11680 | 0.2315 |
| Shaanxi | 26 | 2008 | 95.5 | 97.1 | 94.6 | 8.425529937 | 18246 | 10.29 | 12857.89 | 183510 | 48.4583 |
| Gansu | 36 | 2008 | 91.7 | 83.6 | 87.9 | 7.121462582 | 12110 | 13.22 | 10969.41 | 104179 | 22.2299 |
| Qinghai | 35 | 2008 | 80.4 | 86.1 | 81.9 | 7.181132888 | 17389 | 14.49 | 11640.43 | 25568 | 15.3116 |
| Ningxia | 29 | 2008 | 98.8 | 94 | 97 | 8.051422964 | 17892 | 14.31 | 12931.53 | 31571 | 15.1836 |
| Xinjiang | 43 | 2008 | 91.3 | 94.2 | 82.8 | 8.458766419 | 19893 | 16.05 | 11432.1 | 130174 | 49.1002 |
| Beijing | 8 | 2007 | 98.96 | 99.33 | 96.84 | 10.7840389 | 58204 | 8.32 | 21988.71 | 183050 | 6.7789112 |
| Tianjin | 11 | 2007 | 99.78 | 99.78 | 75.22 | 9.650309972 | 46122 | 7.91 | 16357.35 | 81570 | 8.3227515 |
| Hebei | 20 | 2007 | 93.03 | 95.77 | 88.88 | 8.125436308 | 19877 | 13.33 | 11690.47 | 298945 | 115.5186778 |
| Shanxi | 19 | 2007 | 86.2 | 91.07 | 79.57 | 8.706424839 | 16945 | 11.3 | 11564.95 | 175088 | 152.7625885 |
| Inner Mongolia | 22 | 2007 | 93.5 | 96.73 | 90.64 | 8.282265774 | 25393 | 10.21 | 12377.84 | 126155 | 86.4526055 |
| Liaoning | 11 | 2007 | 97.26 | 98.99 | 95.58 | 8.887059948 | 25729 | 6.89 | 12300.39 | 272720 | 112.9027744 |
| Jilin | 13 | 2007 | 74.8 | 98.06 | 72.7 | 8.701481296 | 19383 | 7.55 | 11285.52 | 160724 | 49.2280204 |
| Heilongjiang | 19 | 2007 | 89.7 | 95.85 | 83.24 | 8.633412675 | 18478 | 7.88 | 10245.28 | 200346 | 64.9296124 |
| Shanghai | 9 | 2007 | 83.21 | 99.27 | 79.78 | 10.24118773 | 66367 | 9.07 | 23622.73 | 155809 | 11.4412493 |
| Jiangsu | 12 | 2007 | 97.06 | 99.58 | 92.42 | 8.351927623 | 33928 | 9.37 | 16378.01 | 355668 | 64.0237126 |
| Zhejiang | 13 | 2007 | 97.96 | 99.45 | 96.97 | 8.019733505 | 37411 | 10.38 | 20573.82 | 273823 | 38.5255315 |
| Anhui | 22 | 2007 | 72.12 | 92.46 | 62.27 | 7.205642381 | 12045 | 12.75 | 11473.58 | 214121 | 61.3199852 |
| Fujian | 13 | 2007 | 95.64 | 98.94 | 91.4 | 7.690120199 | 25908 | 11.9 | 15506.05 | 113467 | 30.4914724 |
| Jiangxi | 28 | 2007 | 89.37 | 94.84 | 88.54 | 8.174494991 | 12633 | 13.86 | 11451.69 | 153238 | 53.226595 |
| Shandong | 16 | 2007 | 97.93 | 99 | 96.73 | 8.167698158 | 27807 | 11.11 | 14264.7 | 401946 | 76.6968234 |
| Henan | 21 | 2007 | 87.29 | 93.92 | 77.87 | 8.142220001 | 16012 | 11.26 | 11477.05 | 384844 | 112.7564936 |
| Hubei | 20 | 2007 | 94.62 | 97.14 | 91.84 | 8.341791495 | 16206 | 9.19 | 11485.8 | 278205 | 52.260793 |
| Hunan | 17 | 2007 | 92.77 | 94.88 | 90.22 | 8.357974739 | 14492 | 11.96 | 12293.54 | 268683 | 110.1786533 |
| Guangdong | 12 | 2007 | 92.2 | 91.71 | 92.38 | 8.615598264 | 33151 | 11.96 | 17699.3 | 452061 | 52.3756158 |
| Guangxi | 22 | 2007 | 93.97 | 92.73 | 89.97 | 7.992354969 | 12555 | 14.19 | 12200.44 | 178396 | 74.4155563 |
| Hainan | 27 | 2007 | 86.38 | 95.21 | 72.05 | 8.262081524 | 14555 | 14.62 | 10996.87 | 40941 | 2.0913828 |
| Chongqing | 23 | 2007 | 84.46 | 84.42 | 79.92 | 7.686268068 | 14660 | 10.1 | 12590.78 | 103563 | 38.0091676 |
| Sichuan | 29 | 2007 | 88.79 | 81.42 | 84.46 | 7.392586209 | 12893 | 9.21 | 11098.28 | 311364 | 65.3646955 |
| Guizhou | 39 | 2007 | 90.29 | 62.89 | 87.29 | 6.810899746 | 6915 | 13.28 | 10678.4 | 101094 | 44.0927946 |
| Yunnan | 31 | 2007 | 93.16 | 74.34 | 89.73 | 6.744805415 | 10540 | 13.08 | 11496.11 | 149403 | 34.7771246 |
| Tibet | 57 | 2007 | 66.13 | 43.26 | 50.3 | 4.605581008 | 12109 | 16.4 | 11130.93 | 10152 | 0.2179032 |
| Shaanxi | 28 | 2007 | 95.81 | 95.01 | 93.93 | 8.32312259 | 14607 | 10.21 | 10763.34 | 177666 | 61.564545 |
| Gansu | 38 | 2007 | 91.03 | 79.11 | 86.57 | 7.026105842 | 10346 | 13.14 | 10012.34 | 101796 | 23.1545368 |
| Qinghai | 38 | 2007 | 85.69 | 84.71 | 85.76 | 7.107929045 | 14257 | 14.93 | 10276.06 | 23936 | 15.0751268 |
| Ningxia | 31 | 2007 | 97.98 | 88.29 | 96.43 | 7.747703733 | 14649 | 14.8 | 10859.33 | 30657 | 18.7093967 |
| Xinjiang | 45 | 2007 | 89.07 | 89.25 | 78.74 | 8.421610169 | 16999 | 16.79 | 10313.44 | 127621 | 47.707602 |
| Beijing | 8 | 2006 | 99.2 | 99.4 | 96.2 | 10.65654732 | 50467 | 6.26 | 19977.52 | 166276 | 8 |
| Tianjin | 11 | 2006 | 81.6 | 99.8 | 76.8 | 9.577022375 | 41163 | 7.67 | 14283.09 | 78631 | 9 |
| Hebei | 22 | 2006 | 92 | 94.3 | 87.7 | 8.090785361 | 16962 | 12.82 | 10304.56 | 279663 | 136.9 |
| Shanxi | 20 | 2006 | 85.9 | 83.9 | 78.3 | 8.630052874 | 14123 | 11.48 | 10027.7 | 178994 | 170.4 |
| Inner Mongolia | 23 | 2006 | 92.4 | 93.5 | 89.6 | 8.127088391 | 20053 | 9.87 | 10357.99 | 120575 | 93 |
| Liaoning | 12 | 2006 | 96.8 | 98.7 | 94.9 | 8.826668439 | 21788 | 6.4 | 10369.61 | 273374 | 113.4 |
| Jilin | 13 | 2006 | 81.9 | 96.7 | 80.5 | 8.589498608 | 15720 | 7.67 | 9775.07 | 161438 | 54.4 |
| Heilongjiang | 19 | 2006 | 88.8 | 91.8 | 82.5 | 8.470863776 | 16195 | 7.57 | 9182.31 | 191945 | 66.4 |
| Shanghai | 9 | 2006 | 83.8 | 99.2 | 80.4 | 10.21993256 | 57695 | 7.47 | 20667.91 | 138002 | 12.3 |
| Jiangsu | 13 | 2006 | 95.5 | 99.5 | 90.9 | 8.180286538 | 28814 | 9.36 | 14084.26 | 334508 | 73.2 |
| Zhejiang | 14 | 2006 | 97.3 | 99.4 | 95.6 | 7.976001669 | 31874 | 10.29 | 18265.1 | 255057 | 42.6 |
| Anhui | 23 | 2006 | 71.1 | 89.7 | 63.5 | 7.290062346 | 10055 | 12.6 | 9771.05 | 204498 | 71.9 |
| Fujian | 14 | 2006 | 95.3 | 98.5 | 91.2 | 7.667957059 | 21471 | 12 | 13753.28 | 124909 | 32.2 |
| Jiangxi | 30 | 2006 | 90.1 | 91.4 | 88.8 | 7.664607408 | 10798 | 13.8 | 9551.12 | 142682 | 57.8 |
| Shandong | 17 | 2006 | 97.5 | 99 | 96.2 | 8.036394225 | 23794 | 11.6 | 12192.24 | 395897 | 90.7 |
| Henan | 23 | 2006 | 84.1 | 90.6 | 74 | 8.012641252 | 13313 | 11.59 | 9810.26 | 374924 | 136 |
| Hubei | 21 | 2006 | 92.8 | 93.6 | 90.2 | 8.180870705 | 13296 | 9.08 | 9802.65 | 265298 | 63.3 |
| Hunan | 18 | 2006 | 91.6 | 91.7 | 89.8 | 8.118072289 | 11950 | 11.92 | 10504.67 | 248018 | 122.5 |
| Guangdong | 13 | 2006 | 91.4 | 89.7 | 91.9 | 8.380784908 | 28332 | 11.78 | 16015.58 | 408972 | 55.9 |
| Guangxi | 24 | 2006 | 92.4 | 88.5 | 87.5 | 7.988250358 | 10296 | 14.44 | 9898.75 | 162725 | 91.9 |
| Hainan | 28 | 2006 | 82.7 | 90.6 | 68 | 8.114719528 | 12654 | 14.59 | 9395.13 | 38199 | 2.1 |
| Chongqing | 25 | 2006 | 86.5 | 78.1 | 79.5 | 7.528447327 | 12457 | 9.9 | 11569.74 | 96742 | 41.4 |
| Sichuan | 31 | 2006 | 83.8 | 76.4 | 82.5 | 7.197167999 | 10546 | 9.14 | 9350.11 | 285785 | 94.1 |
| Guizhou | 42 | 2006 | 89.2 | 54.5 | 86.8 | 6.566778618 | 5787 | 13.97 | 9116.61 | 95654 | 41.7 |
| Yunnan | 34 | 2006 | 92.2 | 68.4 | 88.9 | 6.631537554 | 8970 | 13.2 | 10069.89 | 145621 | 36.6 |
| Tibet | 61 | 2006 | 58 | 37.7 | 51.3 | 4.149936468 | 10430 | 17.4 | 8941.08 | 10746 | 0.2 |
| Shaanxi | 30 | 2006 | 94.3 | 91.5 | 92.3 | 8.224295646 | 12138 | 10.19 | 9267.7 | 168190 | 65.1 |
| Gansu | 40 | 2006 | 89.2 | 73.8 | 83.8 | 6.74435591 | 8757 | 12.86 | 8920.59 | 99431 | 32 |
| Qinghai | 40 | 2006 | 84.2 | 78.2 | 83 | 6.933204881 | 11762 | 15.24 | 9000.35 | 23509 | 16.1 |
| Ningxia | 33 | 2006 | 96.8 | 80.8 | 94.6 | 7.552455796 | 11847 | 15.53 | 9177.26 | 27852 | 20.2 |
| Xinjiang | 47 | 2006 | 85.9 | 81.5 | 75.3 | 8.208427651 | 15000 | 15.79 | 8871.27 | 121400 | 45.6 |
| Beijing | 8 | 2005 | 98.9 | 99.5 | 96.7 | 10.44091644 | 45444 | 6.29 | 17652.95 | 156969 | 9.1 |
| Tianjin | 12 | 2005 | 80.1 | 99.6 | 75.5 | 9.372055516 | 35783 | 7.44 | 12638.55 | 77580 | 11 |
| Hebei | 22 | 2005 | 91.2 | 92.2 | 86.6 | 8.122180624 | 14782 | 12.84 | 9107.09 | 274424 | 144.6 |
| Shanxi | 21 | 2005 | 87.9 | 79.8 | 81.6 | 8.361440085 | 12495 | 12.02 | 8913.91 | 173902 | 181.7 |
| Inner Mongolia | 24 | 2005 | 92.8 | 90.9 | 89.4 | 8.144070693 | 16331 | 10.08 | 9136.79 | 121180 | 123.5 |
| Liaoning | 13 | 2005 | 96.7 | 97.7 | 95.1 | 8.662668733 | 18983 | 7.01 | 9107.55 | 265194 | 119.8 |
| Jilin | 14 | 2005 | 83.6 | 94.7 | 81.5 | 8.401526427 | 13348 | 7.89 | 8690.62 | 157021 | 55 |
| Heilongjiang | 20 | 2005 | 89.3 | 90.8 | 84.4 | 8.395787741 | 14434 | 7.87 | 8272.51 | 191172 | 67.2 |
| Shanghai | 9 | 2005 | 89.2 | 99.4 | 86.3 | 9.847284809 | 51474 | 7.04 | 18645.03 | 132004 | 12.7 |
| Jiangsu | 14 | 2005 | 89.9 | 98.3 | 86.4 | 8.065950401 | 24560 | 9.24 | 12318.57 | 316054 | 80.7 |
| Zhejiang | 16 | 2005 | 96.9 | 99.4 | 95.5 | 7.559495449 | 27703 | 11.1 | 16293.77 | 236197 | 44.3 |
| Anhui | 25 | 2005 | 74.8 | 86 | 68.7 | 7.000136561 | 8675 | 12.43 | 8470.68 | 193973 | 76 |
| Fujian | 15 | 2005 | 94.8 | 98.1 | 91.1 | 7.493153368 | 18646 | 11.6 | 12321.31 | 118652 | 32.4 |
| Jiangxi | 33 | 2005 | 91.3 | 88.8 | 90.2 | 7.492799453 | 9440 | 13.79 | 8619.66 | 138697 | 59.6 |
| Shandong | 19 | 2005 | 97.6 | 99 | 95.7 | 7.677456829 | 20096 | 12.14 | 10744.79 | 381760 | 99.2 |
| Henan | 25 | 2005 | 85 | 87.7 | 75.6 | 7.943325401 | 11346 | 11.55 | 8667.97 | 362263 | 163.2 |
| Hubei | 22 | 2005 | 93.1 | 92.3 | 90 | 7.771531505 | 11431 | 8.74 | 8785.94 | 262263 | 66.8 |
| Hunan | 20 | 2005 | 91 | 86.7 | 89.2 | 7.946021737 | 10426 | 11.9 | 9523.97 | 254423 | 130.8 |
| Guangdong | 14 | 2005 | 91.6 | 87.7 | 91.9 | 8.306853803 | 24435 | 11.7 | 14769.94 | 364520 | 60 |
| Guangxi | 26 | 2005 | 90.4 | 84 | 85.8 | 7.619462484 | 8788 | 14.26 | 9286.7 | 158370 | 110.6 |
| Hainan | 30 | 2005 | 80.2 | 87 | 66.1 | 8.054141259 | 10871 | 14.65 | 8123.94 | 37189 | 2.2 |
| Chongqing | 27 | 2005 | 86.5 | 74 | 82.5 | 7.345539197 | 10982 | 9.4 | 10243.46 | 94761 | 42.9 |
| Sichuan | 33 | 2005 | 87.2 | 73.8 | 83.2 | 6.802417493 | 9060 | 9.7 | 8385.96 | 280516 | 117.5 |
| Guizhou | 46 | 2005 | 88.2 | 48.7 | 85.7 | 6.384930175 | 5052 | 14.59 | 8151.13 | 94854 | 55.5 |
| Yunnan | 37 | 2005 | 91.6 | 64.2 | 87.7 | 6.344088044 | 7835 | 14.72 | 9265.9 | 142175 | 38.2 |
| Tibet | 64 | 2005 | 60.1 | 34.1 | 46.8 | 3.729528084 | 9114 | 17.94 | 9431.18 | 10781 | 0.4 |
| Shaanxi | 32 | 2005 | 93.6 | 88.1 | 90.9 | 8.00064656 | 9899 | 10.02 | 8272.02 | 165170 | 73.4 |
| Gansu | 43 | 2005 | 87.5 | 67.8 | 82.6 | 6.817396168 | 7477 | 12.59 | 8086.82 | 97139 | 32.8 |
| Qinghai | 43 | 2005 | 82.3 | 76.2 | 81.6 | 6.686997859 | 10045 | 15.7 | 8057.85 | 22923 | 17 |
| Ningxia | 36 | 2005 | 96.8 | 78.5 | 94 | 7.30674821 | 10239 | 15.93 | 8093.64 | 27560 | 21.4 |
| Xinjiang | 49 | 2005 | 85.7 | 78.4 | 76.4 | 8.115797112 | 13108 | 16.42 | 7990.15 | 117165 | 44.3 |
| Beijing | 9 | 2004 | 98.1 | 99.5 | 94.9 | 10.31967749 | 37058 | 6.1 | 15637.84 | 153367 | 10.6 |
| Tianjin | 12 | 2004 | 93.7 | 99.7 | 88.7 | 9.501533256 | 31550 | 7.31 | 11467.16 | 77548 | 10.4 |
| Hebei | 23 | 2004 | 91.3 | 94.1 | 87.2 | 8.321275499 | 12918 | 11.98 | 7951.31 | 268399 | 144.8 |
| Shanxi | 22 | 2004 | 87.6 | 74.2 | 81.5 | 8.331055778 | 9150 | 12.36 | 7902.86 | 174658 | 176.4 |
| Inner Mongolia | 25 | 2004 | 91.9 | 85.3 | 89 | 8.103514029 | 11305 | 9.53 | 8122.99 | 120253 | 102 |
| Liaoning | 13 | 2004 | 97.2 | 95.6 | 95.3 | 8.755946935 | 16297 | 6.51 | 8007.56 | 270325 | 92.3 |
| Jilin | 15 | 2004 | 85.3 | 92.9 | 83.5 | 8.730734361 | 10932 | 7.39 | 7840.61 | 161507 | 44.7 |
| Heilongjiang | 20 | 2004 | 89.6 | 85.4 | 85.1 | 8.445158755 | 13897 | 7.27 | 7470.71 | 190563 | 64.4 |
| Shanghai | 10 | 2004 | 86.8 | 99.4 | 84 | 9.927642527 | 55307 | 6 | 16682.82 | 130823 | 13.5 |
| Jiangsu | 15 | 2004 | 92.2 | 98.9 | 88.6 | 7.758454106 | 20705 | 9.45 | 10481.93 | 309450 | 76.8 |
| Zhejiang | 17 | 2004 | 96.4 | 99.3 | 94.9 | 7.875973123 | 23942 | 10.71 | 14546.38 | 221539 | 55.2 |
| Anhui | 26 | 2004 | 76.2 | 84.2 | 71.5 | 7.443036683 | 7768 | 11.62 | 7511.43 | 190763 | 71.6 |
| Fujian | 16 | 2004 | 95.6 | 97.3 | 91.8 | 7.446199469 | 17218 | 11.58 | 11175.37 | 117937 | 28.5 |
| Jiangxi | 35 | 2004 | 91.8 | 85.8 | 90.6 | 7.936374269 | 8189 | 13.61 | 7559.64 | 141244 | 57.3 |
| Shandong | 20 | 2004 | 96.8 | 98.8 | 95 | 7.889028913 | 16925 | 12.5 | 9437.8 | 377950 | 97.4 |
| Henan | 26 | 2004 | 83.2 | 85.6 | 72.4 | 8.175033319 | 9470 | 11.67 | 7704.9 | 356925 | 148.7 |
| Hubei | 23 | 2004 | 93.3 | 89.9 | 89.9 | 8.038355428 | 10500 | 8.43 | 8022.75 | 260569 | 65 |
| Hunan | 22 | 2004 | 93.2 | 85.3 | 91.6 | 8.104960673 | 9117 | 11.89 | 8617.48 | 249619 | 125.7 |
| Guangdong | 15 | 2004 | 92.3 | 85.5 | 92.2 | 8.078288331 | 19707 | 13.13 | 13627.65 | 348203 | 65.5 |
| Guangxi | 28 | 2004 | 86.9 | 76 | 83.3 | 7.963953567 | 7196 | 13.32 | 8689.99 | 154384 | 105.9 |
| Hainan | 31 | 2004 | 81.4 | 86.1 | 66.5 | 8.353934114 | 9450 | 14.77 | 7735.78 | 36835 | 2.2 |
| Chongqing | 29 | 2004 | 86.4 | 71.1 | 82.3 | 7.210152781 | 9608 | 9.45 | 9220.96 | 93485 | 42.4 |
| Sichuan | 35 | 2004 | 89.4 | 74.4 | 86.2 | 7.418260989 | 8113 | 9.05 | 7709.87 | 281847 | 130.3 |
| Guizhou | 50 | 2004 | 86.5 | 38.9 | 83.3 | 6.936899229 | 4215 | 15.08 | 7322.05 | 90174 | 57.1 |
| Yunnan | 40 | 2004 | 89.9 | 60.1 | 85.9 | 6.777295697 | 6733 | 15.6 | 8870.88 | 136697 | 30.7 |
| Tibet | 67 | 2004 | 64.9 | 29.8 | 51.8 | 4.38763815 | 7779 | 17.4 | 9106.07 | 10260 | 0.2 |
| Shaanxi | 34 | 2004 | 92.1 | 85.3 | 90.1 | 8.190120418 | 7757 | 10.59 | 7492.47 | 163850 | 72.9 |
| Gansu | 45 | 2004 | 87 | 62.5 | 82.6 | 7.179174012 | 5970 | 12.43 | 7376.74 | 96423 | 31.1 |
| Qinghai | 46 | 2004 | 82.3 | 66.4 | 81.2 | 6.756728562 | 8606 | 16.32 | 7319.67 | 23005 | 15.9 |
| Ningxia | 38 | 2004 | 95.1 | 75.9 | 92.4 | 7.630977735 | 7880 | 15.97 | 7217.87 | 27335 | 18.3 |
| Xinjiang | 51 | 2004 | 83.7 | 69 | 72.1 | 8.386126178 | 11199 | 16 | 7503.42 | 120502 | 42.8 |
| Beijing | 9 | 2003 | 97.7 | 99.28 | 94.34 | 10.14284663 | 32061 | 5.1 | 13882.62 | 148191 | 10.2928 |
| Tianjin | 13 | 2003 | 95.08 | 99.62 | 90.73 | 9.138219895 | 26532 | 7.14 | 10312.91 | 78286 | 12.4627 |
| Hebei | 24 | 2003 | 92.08 | 93.72 | 88.67 | 8.312669496 | 10513 | 11.43 | 7239.06 | 262278 | 135.422 |
| Shanxi | 23 | 2003 | 89.3 | 68.03 | 82.59 | 8.344518721 | 7435 | 12.26 | 7005.03 | 169870 | 173.2043 |
| Inner Mongolia | 26 | 2003 | 87.44 | 79.4 | 84.98 | 7.713784258 | 8975 | 9.24 | 7012.9 | 120369 | 80.3744 |
| Liaoning | 14 | 2003 | 96.93 | 92.53 | 94.43 | 8.830771944 | 14258 | 6.9 | 7240.58 | 269252 | 93.5054 |
| Jilin | 15 | 2003 | 86.69 | 87.96 | 84.04 | 8.638376095 | 9338 | 7.25 | 7005.17 | 161398 | 40.4131 |
| Heilongjiang | 21 | 2003 | 91.04 | 81.62 | 85.23 | 8.360135774 | 11615 | 7.48 | 6678.9 | 192858 | 61.7674 |
| Shanghai | 10 | 2003 | 87.39 | 99.15 | 84.18 | 9.958975666 | 46718 | 4.85 | 14867.49 | 133038 | 13.2405 |
| Jiangsu | 16 | 2003 | 92.09 | 98.25 | 88.34 | 7.640741539 | 16809 | 9.04 | 9262.46 | 302192 | 83.7818 |
| Zhejiang | 18 | 2003 | 97.33 | 99.31 | 96.23 | 7.698295716 | 20147 | 9.66 | 13179.53 | 207937 | 57.3107 |
| Anhui | 27 | 2003 | 85.79 | 84.46 | 81.7 | 7.614295656 | 6455 | 11.15 | 6778.03 | 185916 | 70.1895 |
| Fujian | 17 | 2003 | 96.03 | 96.2 | 93.45 | 7.540334336 | 14979 | 11.43 | 9999.54 | 114893 | 26.9071 |
| Jiangxi | 37 | 2003 | 92.5 | 83.07 | 91.46 | 8.230419889 | 6678 | 14.07 | 6901.42 | 138068 | 52.9781 |
| Shandong | 21 | 2003 | 96.67 | 98.41 | 95.67 | 7.796907807 | 13661 | 11.42 | 8399.91 | 366895 | 138.4311 |
| Henan | 28 | 2003 | 82.47 | 82.89 | 72.18 | 7.934752607 | 7570 | 12.1 | 6926.12 | 348890 | 142.5868 |
| Hubei | 24 | 2003 | 92.76 | 87.22 | 90.88 | 7.865774433 | 9011 | 8.26 | 7321.98 | 255648 | 62.8316 |
| Hunan | 23 | 2003 | 93.26 | 82.93 | 92.23 | 8.004470915 | 7554 | 11.82 | 7674.2 | 254697 | 118.5265 |
| Guangdong | 16 | 2003 | 92.4 | 84.77 | 92.89 | 7.957582583 | 17213 | 13.66 | 12380.43 | 336175 | 66.7244 |
| Guangxi | 30 | 2003 | 87.94 | 72.31 | 85.11 | 7.722713931 | 5969 | 13.86 | 7785.04 | 146747 | 103.259 |
| Hainan | 32 | 2003 | 78.98 | 84.38 | 62.25 | 8.132682133 | 8316 | 14.68 | 7259.25 | 36191 | 2.401 |
| Chongqing | 31 | 2003 | 89.63 | 70.24 | 86.3 | 7.633388567 | 7209 | 9.89 | 8093.67 | 92978 | 43.2833 |
| Sichuan | 38 | 2003 | 86.95 | 69.09 | 82.87 | 7.378460121 | 6418 | 9.18 | 7041.87 | 288300 | 129.9509 |
| Guizhou | 55 | 2003 | 82.26 | 33.03 | 79.9 | 6.83583124 | 3603 | 15.91 | 6569.23 | 91057 | 64.4639 |
| Yunnan | 43 | 2003 | 69.99 | 43.66 | 64.8 | 6.022186842 | 5662 | 17 | 7643.57 | 134508 | 29.2262 |
| Tibet | 70 | 2003 | 64.69 | 26.74 | 47.62 | 3.863149217 | 6871 | 17.4 | 8765.45 | 10058 | 0.2732 |
| Shaanxi | 36 | 2003 | 92.44 | 82.43 | 89.55 | 8.047743737 | 6480 | 10.67 | 6806.35 | 164398 | 66.522 |
| Gansu | 47 | 2003 | 85.14 | 57.91 | 80.22 | 6.993231386 | 5022 | 12.58 | 6657.24 | 96538 | 33.1588 |
| Qinghai | 49 | 2003 | 82.26 | 64.18 | 78.25 | 6.669525204 | 7277 | 16.94 | 6745.32 | 22987 | 11.8242 |
| Ningxia | 40 | 2003 | 91.67 | 69.32 | 87.66 | 7.292145781 | 6691 | 15.68 | 6530.48 | 27589 | 30.6874 |
| Xinjiang | 54 | 2003 | 81.22 | 62.01 | 73.89 | 8.276233495 | 9700 | 16.01 | 7173.54 | 116584 | 36.4335 |
| Beijing | 10 | 2002 | 97.6 | 99.2 | 94.9 | 10.05415817 | 28449 | 6.6 | 12463.92 | 143915 | 7.9551 |
| Tianjin | 13 | 2002 | 96.1 | 86.4 | 91.8 | 9.04442344 | 22380 | 7.49 | 9337.56 | 78112 | 10.3511 |
| Hebei | 25 | 2002 | 92.5 | 91.1 | 90.5 | 7.985795999 | 9115 | 11.53 | 6679.68 | 262202 | 117.5595 |
| Shanxi | 24 | 2002 | 88.2 | 66.1 | 87.5 | 8.199423688 | 6146 | 12.86 | 6234.36 | 166695 | 131.0497 |
| Inner Mongolia | 28 | 2002 | 93.4 | 83.5 | 96.8 | 7.821640583 | 7241 | 9.6 | 6051 | 120608 | 39.2798 |
| Liaoning | 14 | 2002 | 97.3 | 88 | 95.5 | 8.38459018 | 12986 | 7.38 | 6524.52 | 267237 | 76.3487 |
| Jilin | 16 | 2002 | 88.9 | 86.4 | 83.6 | 8.548705882 | 8334 | 8.3 | 6260.16 | 160351 | 32.9366 |
| Heilongjiang | 21 | 2002 | 90.8 | 81.4 | 84 | 8.250312439 | 10184 | 7.98 | 6100.56 | 198545 | 47.6439 |
| Shanghai | 10 | 2002 | 95.6 | 99.4 | 93.4 | 9.444307971 | 40646 | 5.41 | 13249.8 | 132859 | 7.1018 |
| Jiangsu | 17 | 2002 | 92.6 | 97.5 | 88.9 | 7.550275829 | 14391 | 9.17 | 8177.64 | 301404 | 59.5694 |
| Zhejiang | 19 | 2002 | 97.2 | 98.1 | 96.4 | 7.617866062 | 16838 | 9.98 | 11715.6 | 199095 | 51.3324 |
| Anhui | 29 | 2002 | 85.7 | 82.5 | 79.8 | 6.961624131 | 5817 | 11.2 | 6032.4 | 183823 | 44.7972 |
| Fujian | 18 | 2002 | 96.1 | 95.1 | 93.7 | 7.414744108 | 13497 | 11.35 | 9189.36 | 124022 | 22.2321 |
| Jiangxi | 40 | 2002 | 92.7 | 80.4 | 89.2 | 7.450077126 | 5829 | 14.74 | 6335.64 | 142728 | 38.8306 |
| Shandong | 22 | 2002 | 96.7 | 99 | 98 | 8.022758432 | 11645 | 11.17 | 7614.36 | 366814 | 110.5681 |
| Henan | 31 | 2002 | 82.1 | 79.3 | 73.2 | 8.033353004 | 6436 | 12.41 | 6245.4 | 333921 | 130.5541 |
| Hubei | 26 | 2002 | 92.3 | 86.6 | 92.8 | 7.30356319 | 8319 | 8.38 | 6788.52 | 256915 | 60.4918 |
| Hunan | 26 | 2002 | 93.9 | 81.2 | 93.9 | 7.866558151 | 6565 | 11.56 | 6958.56 | 245585 | 97.5522 |
| Guangdong | 17 | 2002 | 92.5 | 85.6 | 91.3 | 8.042870093 | 15030 | 13.29 | 11137.2 | 323416 | 51.3798 |
| Guangxi | 32 | 2002 | 87.9 | 68.1 | 84.9 | 7.586831369 | 5099 | 13.3 | 7315.32 | 149286 | 88.0658 |
| Hainan | 33 | 2002 | 79.2 | 83.5 | 65.3 | 7.906915991 | 7803 | 15.2 | 6822.72 | 36200 | 2.3259 |
| Chongqing | 33 | 2002 | 86.9 | 66.9 | 84 | 7.404919682 | 6347 | 9.36 | 7238.04 | 96087 | 31.3954 |
| Sichuan | 40 | 2002 | 90.7 | 70.8 | 88.7 | 7.249201758 | 5766 | 10.44 | 6610.8 | 292552 | 114.0691 |
| Guizhou | 60 | 2002 | 82 | 31.1 | 79.8 | 6.6971534 | 3153 | 17.96 | 5944.08 | 91209 | 50.3502 |
| Yunnan | 46 | 2002 | 88.7 | 56 | 83.9 | 6.09926327 | 5179 | 17.9 | 7240.56 | 133115 | 21.0033 |
| Tibet | 75 | 2002 | 61.6 | 33.3 | 57 | 4.315461347 | 6093 | 18.83 | 8079.12 | 10275 | 0.2954 |
| Shaanxi | 37 | 2002 | 91.1 | 80.4 | 88.6 | 7.390876435 | 5523 | 10.48 | 6330.84 | 165746 | 53.681 |
| Gansu | 51 | 2002 | 85.2 | 58.5 | 79.9 | 6.749968531 | 4493 | 13.16 | 6151.44 | 95495 | 23.2343 |
| Qinghai | 52 | 2002 | 79.1 | 57.3 | 76.5 | 6.316892464 | 6426 | 18.05 | 6170.52 | 23506 | 7.972 |
| Ningxia | 43 | 2002 | 93.8 | 70.4 | 91.3 | 7.335438043 | 5804 | 16.42 | 6067.44 | 25590 | 21.1605 |
| Xinjiang | 57 | 2002 | 82.3 | 62.3 | 78.9 | 8.267618002 | 8382 | 16.3 | 6899.64 | 110771 | 19.1642 |

# supplementary file2

**Control Variable Definition**

**Total number of health workers** are from the China Health Statistics Yearbook. Health workers refer to employees working in hospitals, primary health care institutions, professional public health institutions and other health care institutions, including health technicians, rural doctors and health workers, other technicians, managers and workers. That is, the statistics of the number of employees on duty who paid their salaries at the end of the year, including all types of employed staff (including contract workers) and those who have returned to the unit for more than six months, excluding temporary workers, retired staff, retired staff, those who have left the unit and still retain their labor relations, and those who have returned to the unit and have been temporarily employed for less than six months.

**Birth rate** are from the China Statistical Yearbook. The ratio of the number of births in a certain area in a certain period (usually one year) to the average number (or mid-period number) in the same period, expressed in thousands. The birth rate in this information refers to the annual birth rate, which is calculated as $Birth rate=\frac{the number of births}{the average number \mathrm{of} \mathrm{people}}*1000‰$。. Where: The number of births refers to live births, i.e., when the fetus was detached from the mother (regardless of the number of months of pregnancy), there was breathing or other signs of life. The average number of people refers to the average number of people at the beginning and end of the year, and can also be replaced by the mid-year population.

**GDP per capita** are from the China Statistical Yearbook. GDP refers to the final products produced by all resident units in a country during a certain period of time. Gross domestic product is expressed in three different perspectives, namely value, income, and products respectively. GDP in its value perspective refers to the balance of total value of all goods and services produced by all resident units during a certain period of time, minus the total value of input of goods and services of the nature of non-fixed assets; in other words, it is the sum of the value-added of all resident units. GDP from the perspective of income refers to the sum of all kinds of revenue, including compensation of employees, net taxes on production, depreciation of fixed assets, and operating surplus. GDP from the perspective of products refers to the value of all goods ang services for the final demand by all resident units plus the net exports of goods and services during a given period of time. In the practice of national accounting, gross domestic product is calculated by three approaches, namely production approach, income approach and expenditure approach, which reflect gross domestic product and its composition from different angles. For a region, it is called as gross domestic product (GRP) or regional GDP.

**Disposable income per capita** are from the China Statistical Yearbook. Disposable Income of Residents refers to the income of residents for purpose of final expenditure and savings. It includes income both in cash and in kind. By sources of income, disposable income includes four categories: income from wages and salaries, net business income, net income from properties and net income from transfer.

**Years of education per capita** are from the China Statistical Yearbook. The number of people aged 6 years and above and the number of people by education level in each region were collected from the China Statistical Yearbook, and the formula was calculated as

$$the average years of education for people aged 6 years and above= (elementary school \times6 + middle school \times9 + high school \times12 + secondary school \times12 + college \times15 + undergraduate \times16 + graduate \times19) \div total number of people aged 6 years and above$$

Calculation method reference: Hu Angang, Li Chunbo. New Poverty in the New Century:Knowledge Poverty[J]. China Social Science,2001(03):70-81+206.

**Emissions of particulate matter in exhaust gases** are from the China Statistical Yearbook. Exhaust gas refers to the toxic and harmful gases emitted by human beings in the process of production and living. Especially chemical plants, iron and steel plants, pharmaceutical plants, as well as coke plants and oil refineries, etc., emit exhaust gases with high odor, which seriously pollute the environment and affect human health. China's Environmental Protection Law has clearly defined the emission standards for various types of factories and mines. Particulate matter, also known as dust, is a variety of solid or liquid particles that are uniformly dispersed in an aerosol system. Particulate matter can be divided into primary and secondary particulate matter. Primary particulate matter is particulate matter that is released into the atmosphere from direct sources that cause pollution, such as soil particles, sea salt particles, combustion soot, etc. Secondary particulate matter is particulate matter generated by the conversion of certain pollutant gas components in the atmosphere (such as sulfur dioxide, nitrogen oxides, hydrocarbons, etc.) to each other or between these components and normal components of the atmosphere (such as oxygen) through photochemical oxidation reactions, catalytic oxidation reactions, or other chemical reactions, such as the conversion of sulfur dioxide to sulfate.

# supplementary file3

**WHO Definitions of Inequity Measurement Indicators**

**Difference (D)**

**Definition**

D is an absolute measure of inequality that shows the difference between two population subgroups. It is calculated for all inequality dimensions, provided that subgroup estimates are available for the two subgroups used in the calculation of D.

**Calculation**

D is calculated as the difference between two population subgroups:

D= 𝑦_ℎ𝑖𝑔ℎ_ −𝑦_𝑙𝑜𝑤_

Note that the selection of 𝑦_ℎ𝑖𝑔ℎ_ and 𝑦_𝑙𝑜𝑤_ depends on the characteristics of the inequality dimension and the type of indicator, for which D is calculated. Table 1 provides an overview of the calculation of D.

Table 1 provides an overview of the calculation of D.


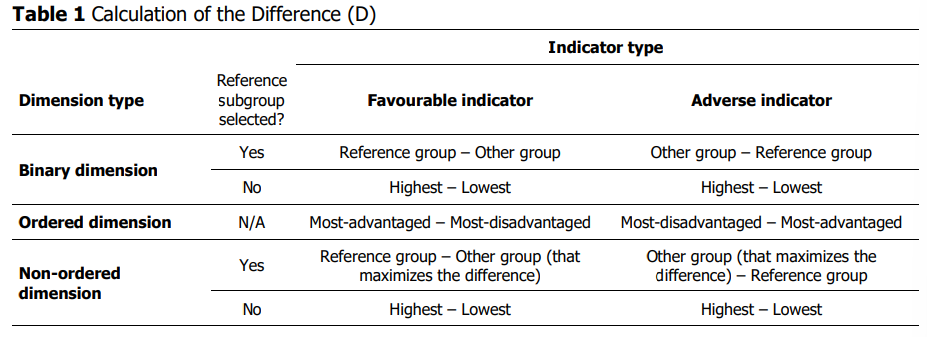


**Interpretation**

If there is no inequality, D takes the value zero. Greater absolute values indicate higher levels of inequality.

**Absolute concentration index (ACI)**

**Definition**

ACI shows the gradient across population subgroups, on an absolute scale. It indicates the extent to which an indicator is concentrated among disadvantaged or advantaged subgroups.

ACI is an absolute measure of inequality that takes into account all population subgroups. It is calculated for ordered dimensions with more than two subgroups, such as economic status. Subgroups are weighted according to their population share. ACI is missing if at least one subgroup estimates or subgroup population share is missing.

**Calculation**

The calculation of ACI is based on a ranking of the whole population from the most-disadvantaged subgroup (at rank 0) to the most-advantaged subgroup (at rank 1), which is inferred from the ranking and size of the subgroups. The relative rank of each subgroup is calculated as: $X_{j}=\sum jp_{j}-0.5p_{j}$. Based on this ranking, ACI can be calculated as:

$$ACI=\sum_{j} p_{j}(2X_{j}-1)y_{j}$$

where 𝑦_𝑗_ indicates the estimate for subgroup j, 𝑝_𝑗_ the population share of subgroup j and 𝑋_𝑗_ the relative rank of subgroup j.

**Interpretation**

If there is no inequality, ACI takes the value zero. Positive values indicate a concentration of the indicator among the advantaged, while negative values indicate a concentration of the indicator among the disadvantaged. The larger the absolute value of ACI, the higher the level of inequality.

**Population attributable fraction (PAF)**

**Definition**

PAF shows the potential for improvement in setting average of an indicator, in relative terms, that could be achieved if all population subgroups had the same level of the indicator as a reference group.

PAF is a relative measure of inequality that takes into account all population subgroups. It is calculated for all inequality dimensions, provided that all subgroup estimates and subgroup population shares are available.

**Calculation**

PAF is calculated by dividing the population attributable risk (PAR) by the setting average 𝜇 and multiplying the fraction by 100:

$$PAF=\frac{PAR}{\mu}*100$$

**Interpretation**

PAF takes positive values for favorable indicators and negative values for adverse indicators. The larger the absolute value of PAF, the larger the level of inequality. PAF is zero if no further improvement can be achieved, i.e., if all subgroups have reached the same level of the indicator as the reference subgroup.

**Population attributable risk (PAR)**

**Definition**

PAR shows the potential for improvement in setting average that could be achieved if all population subgroups had the same level of the indicator as a reference group.

PAR is an absolute measure of inequality that takes into account all population subgroups. It is calculated for all inequality dimensions, provided that all subgroup estimates and subgroup population shares are available.

**Calculation**

PAR is calculated as the difference between the estimate for the reference subgroup 𝑦𝑟𝑒𝑓 and the setting average μ:

𝑃𝐴𝑅 = 𝑦_𝑟𝑒𝑓_ – μ

Note that the reference subgroup 𝑦𝑟𝑒𝑓 depends on the characteristics of the inequality dimension and indicator type, for which PAR is calculated. Table 2 provides an overview of the calculation of PAR.


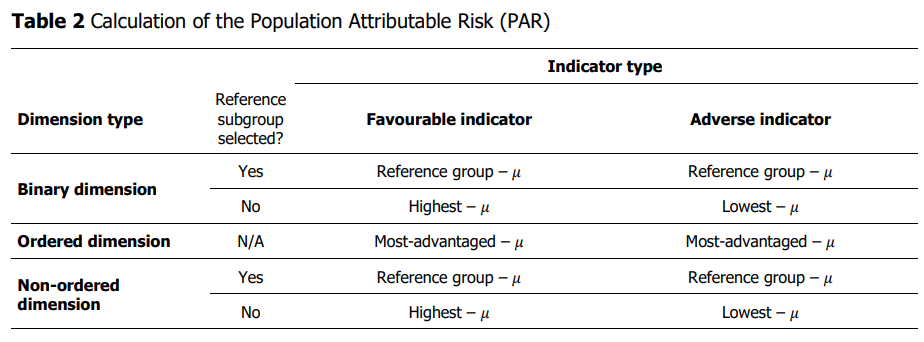


**Interpretation**

PAR takes positive values for favorable indicators and negative values for adverse indicators. The larger the absolute value of PAR, the higher the level of inequality. PAR is zero if no further improvement can be achieved, i.e., if all subgroups have reached the same level of the indicator as the reference subgroup.

**Ratio (R)**

**Definition**

R is a relative measure of inequality that shows the ratio of two population subgroups. It is calculated for all inequality dimensions, provided that subgroup estimates are available for the two subgroups used in the calculation of R.

**Calculation**

R is calculated as the ratio of two subgroups:

𝑅 = 𝑦_ℎ𝑖𝑔ℎ_⁄𝑦_𝑙𝑜𝑤_

Note that the selection of 𝑦ℎ𝑖𝑔ℎ and 𝑦𝑙𝑜𝑤 depends on the characteristics of the inequality dimension and the type of indicator, for which R is calculated. Table 3 provides an overview of the calculation of R.


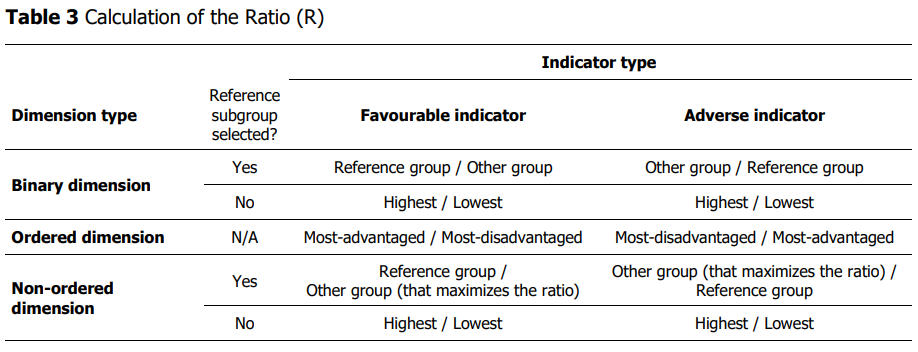


R is calculated for all dimensions of inequality. In the case of binary and non-ordered dimensions, R is missing if at least one subgroup estimate is missing. In the case of ordered dimensions, R is missing if the estimates for the most-advantaged and/or most-disadvantaged subgroup are missing.

**Interpretation**

If there is no inequality, R takes the value one. R takes only positive values. The further the value of R from one, the higher the level of inequality.

Note that R is displayed on a logarithmic scale. R values are intrinsically asymmetric: a ratio of one (no inequality) is halfway between a ratio of 0.5 (the denominator subgroup having half the value of the numerator subgroup) and a ratio of 2.0 (the denominator subgroup having double the value of the numerator subgroup). On a regular axis, R values would be concentrated at the lower end of the scale, with a few very large outlier values at the upper end of the scale. On a logarithmic axis, these values are equally spaced, making them easier to read and interpret.

**Relative concentration index (RCI)**

**Definition**

RCI shows the gradient across population subgroups, on a relative scale. It indicates the extent to which an indicator is concentrated among disadvantaged or advantaged subgroups.

RCI is a relative measure of inequality that takes into account all population subgroups. It is calculated for ordered dimensions with more than two subgroups, such as economic status. Subgroups are weighted according to their population share. RCI is missing if at least one subgroup estimates or subgroup population share is missing

**Calculation**

RCI is calculated by dividing the absolute concentration index (ACI) by the setting average 𝜇 and multiplying the fraction by 100:

$$RCI=\frac{ACI}{\mu}*100$$

**Interpretation**

RCI is bounded between -100 and +100 and takes the value zero if there is no inequality. Positive values indicate a concentration of the indicator among the advantaged, while negative values indicate a concentration of the indicator among the disadvantaged. The greater the absolute value of RCI, the higher the level of inequality.

**Slope index of inequality (SII)**

**Definition**

SII represents the difference in estimated values of an indicator between the most-advantaged and most-disadvantaged (or vice versa for adverse indicators), while taking into consideration all the other subgroups – using an appropriate regression model.

SII is an absolute measure of inequality that takes into account all population subgroups. It is calculated for ordered dimensions with more than two subgroups, such as economic status. Subgroups are weighted according to their population share. SII is missing if at least one subgroup estimates or subgroup population share is missing.

**Calculation**

To calculate SII, a weighted sample of the whole population is ranked from the most-disadvantaged subgroup (at rank 0) to the most-advantaged subgroup (at rank 1). This ranking is weighted, accounting for the proportional distribution of the population within each subgroup. The population of each subgroup is then considered in terms of its range in the cumulative population distribution, and the midpoint of this range. According to the definition currently used in HEAT, the indicator of interest is then regressed against this midpoint value using a generalized linear model with logit link, and the predicted values of the indicator are calculated for the two extremes (rank 1 and rank 0).

For **favorable indicators**, the difference between the estimated values at rank 1 (𝑣_1_) and rank 0 (𝑣_0_) (covering the entire distribution) generates the SII value:

𝑆𝐼𝐼 = 𝑣_1_ −𝑣_0_

For **adverse indicators**, the calculation is reversed and the SII value is calculated as the difference between the estimated values at rank 0 (𝑣_0_) and rank 1 (𝑣_1_) (covering the entire distribution):

𝑆𝐼𝐼 = 𝑣_0_ − 𝑣_1_

**Interpretation**

If there is no inequality, SII takes the value zero. Greater absolute values indicate higher levels of inequality. For favorable indicators, positive values indicate a concentration of the indicator among the advantaged and negative values indicate a concentration of the indicator among the disadvantaged. For adverse indicators, positive values indicate a concentration of the indicator among the disadvantaged and negative values indicate a concentration of the indicator among the advantaged.

# supplementary file4

**Trends in U5MR by province in China, 1990-2017**


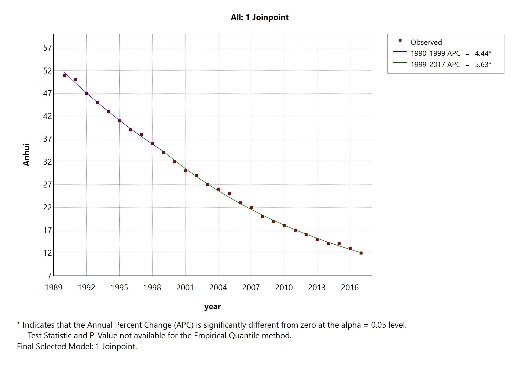

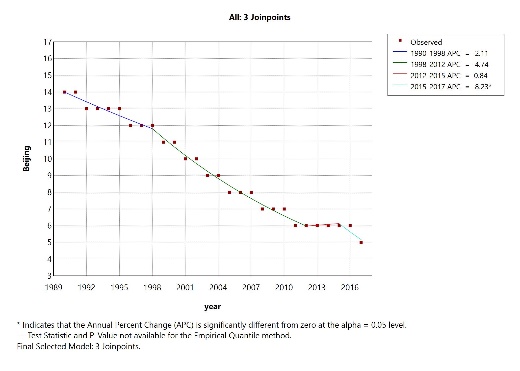


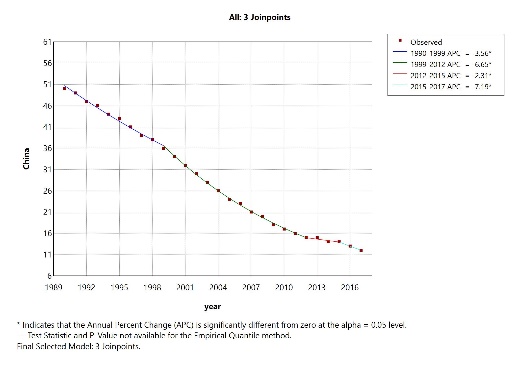

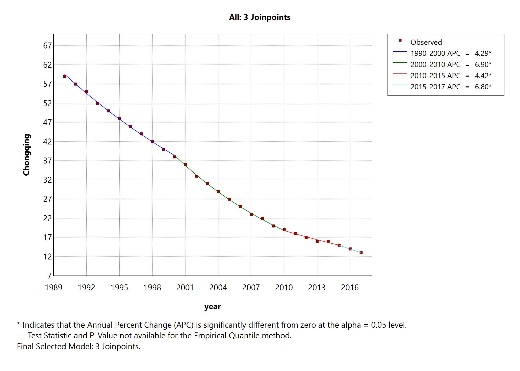


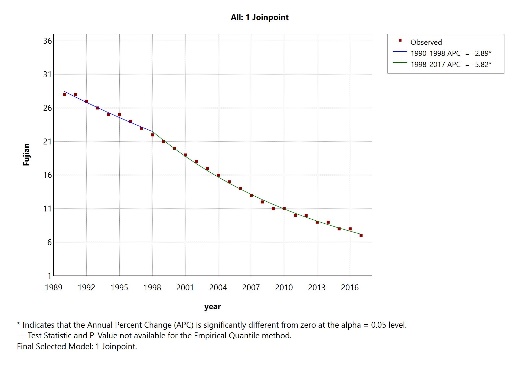

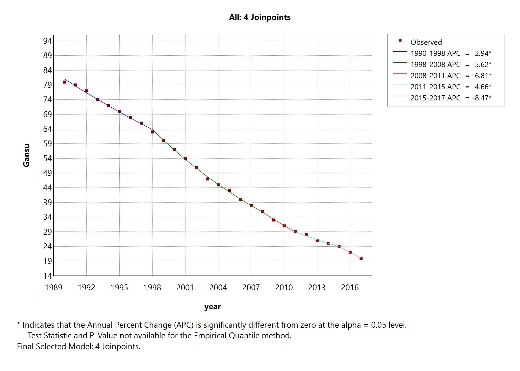


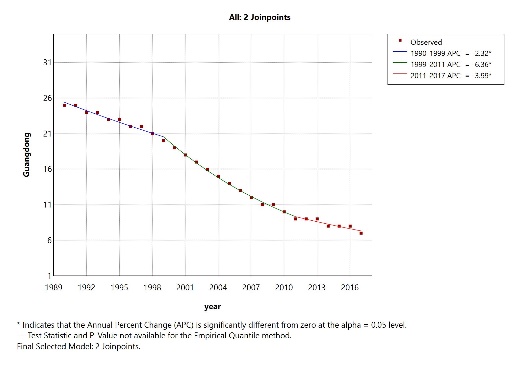

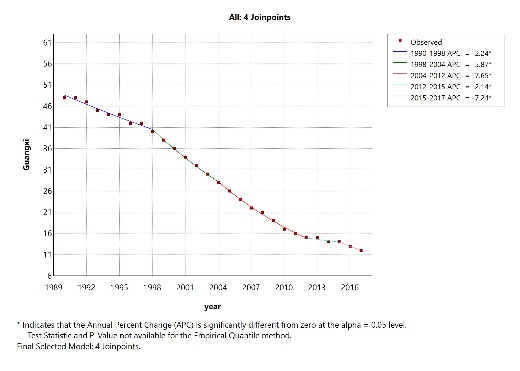


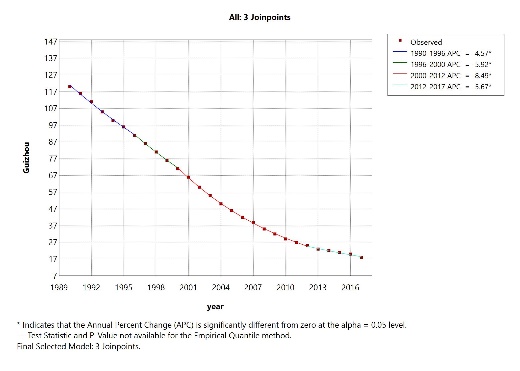

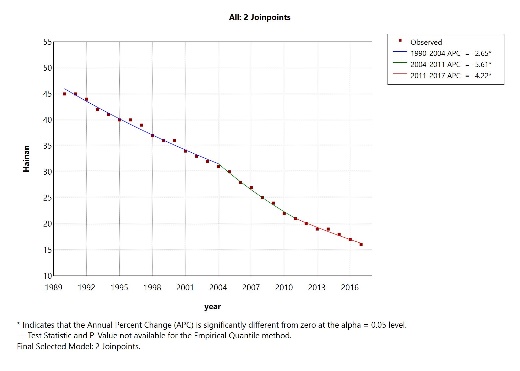


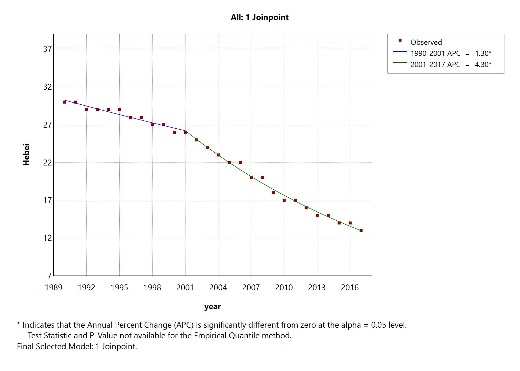

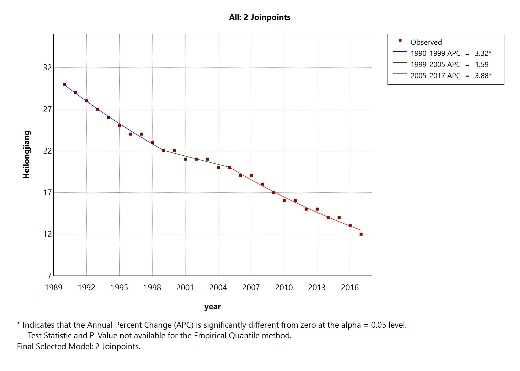


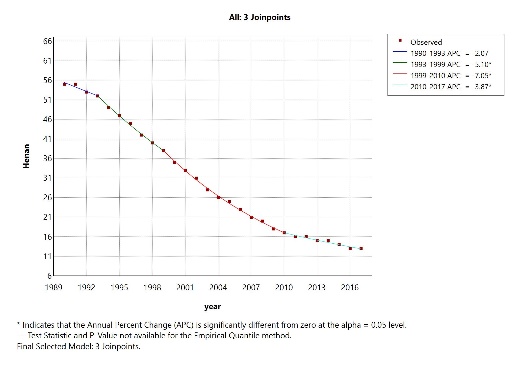

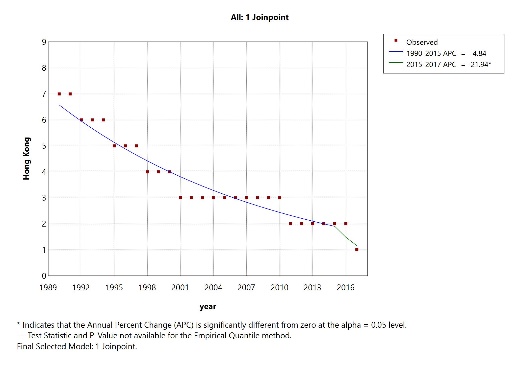


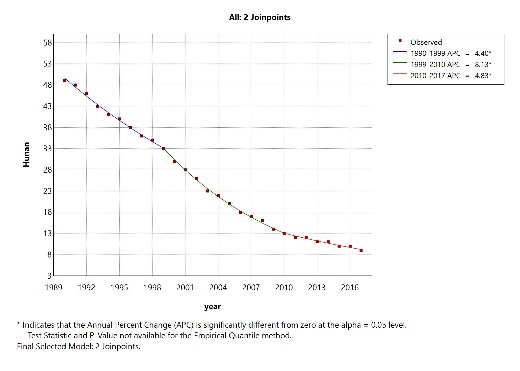

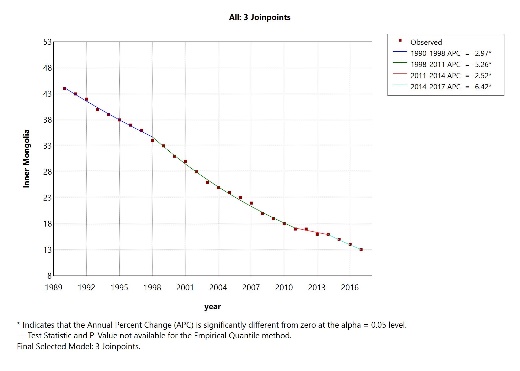


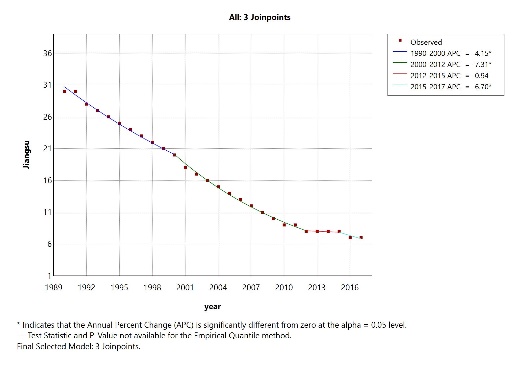

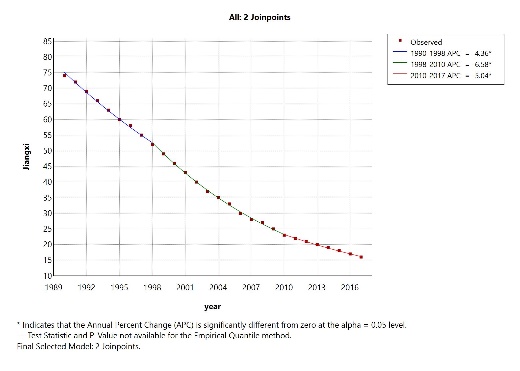


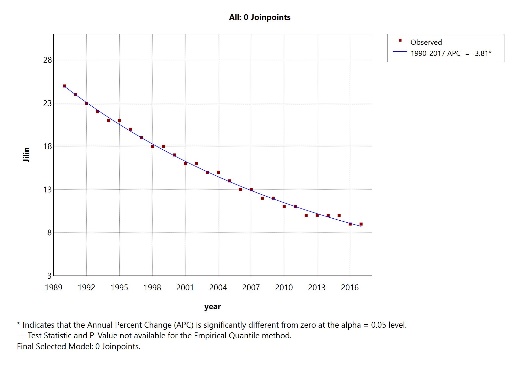

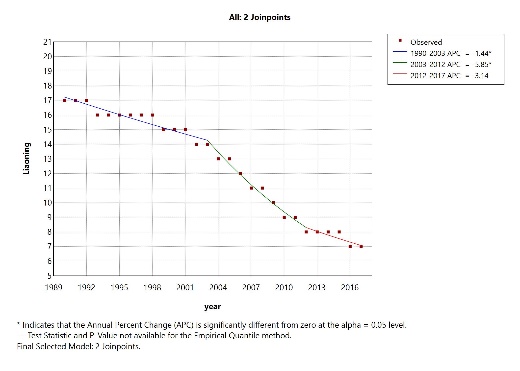


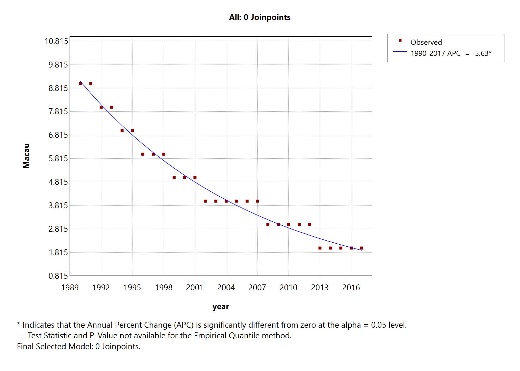

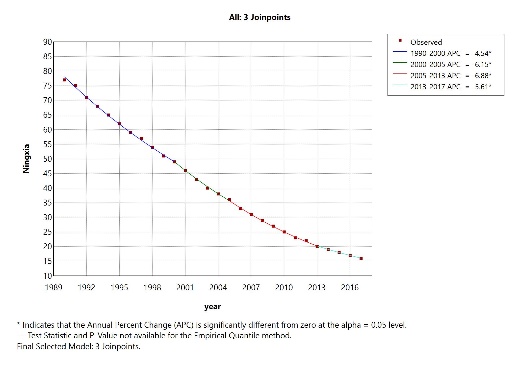


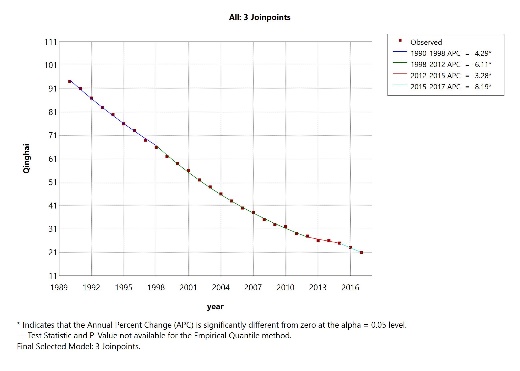

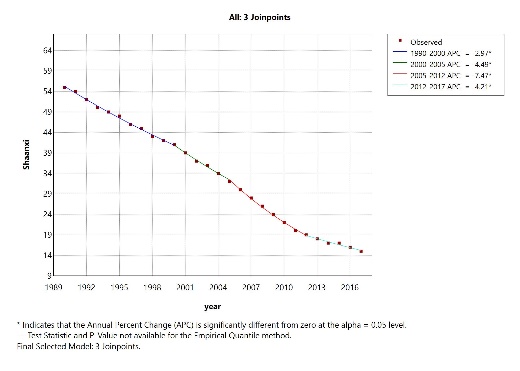


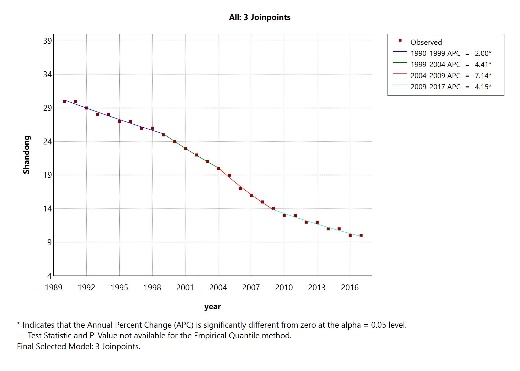

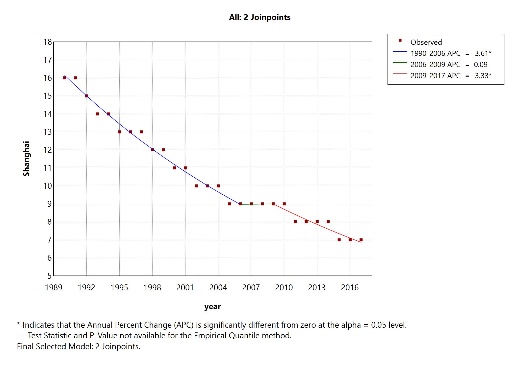


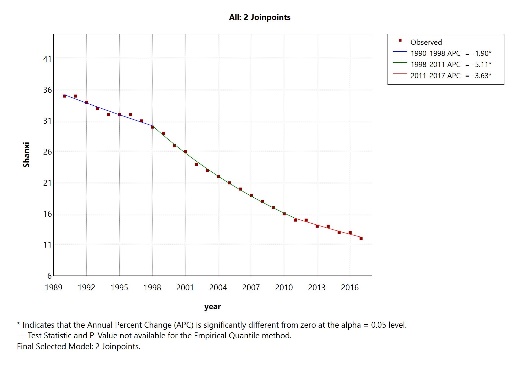

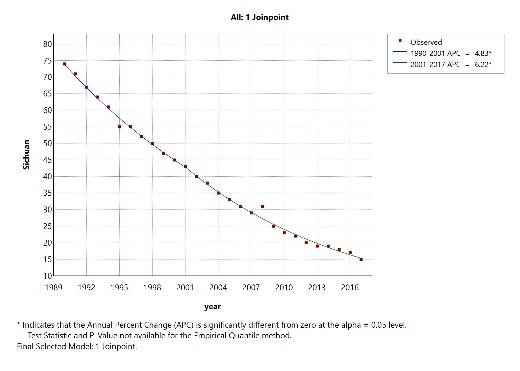


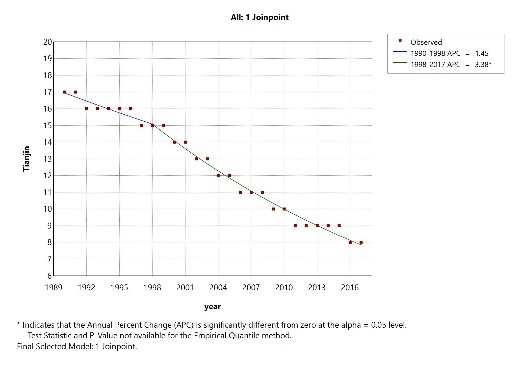

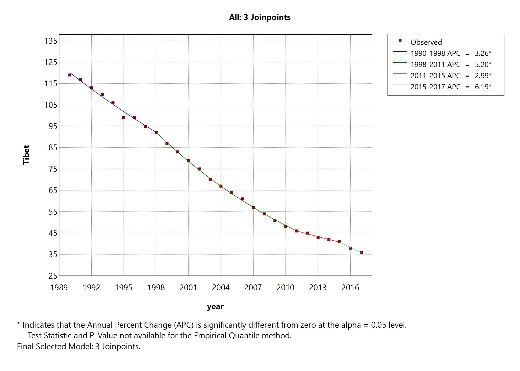


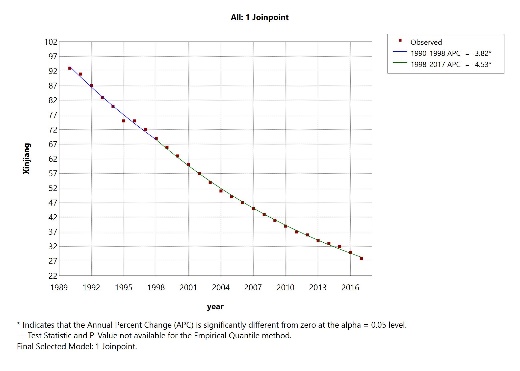

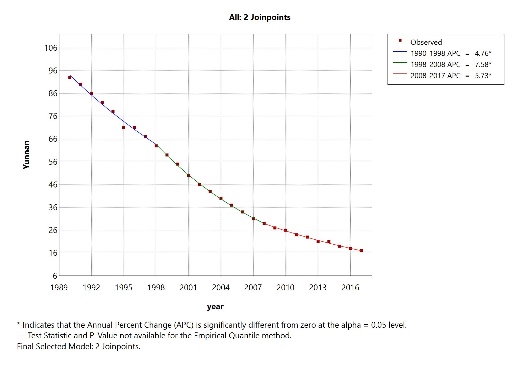


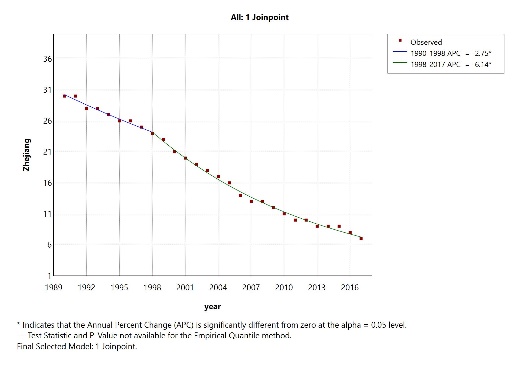

Supplement: Supplementary file 1 — Supplementary Material 1 [file 12884_2024_6437_MOESM1_ESM.docx]
